# A 3D Printing Strategy for Hard Curved Surfaced Circuits: From Materials to Applications

**DOI:** 10.1002/advs.202417648

**Published:** 2025-08-28

**Authors:** Wang Qin, Shujuan Li, Wei Wei, Haiqing Bai, Shikui Jia, Robert G Landers, Yunlong Ma, Sha Wei, Tuo Kang, Miao Zhang, Weipei Zhang, Mingkun Wang, Tongxin Guan

**Affiliations:** ^1^ School of Mechanical and Precision Instrument Engineering Xi'an University of Technology Xi'an Shaanxi 710000 China; ^2^ School of Mechanical Engineering Shaanxi University of Technology Hanzhong Shaanxi 723001 China; ^3^ Shaanxi Key Laboratory of Industrial Automation Hanzhong Shaanxi 723001 China; ^4^ School of Materials Science and Engineering Shaanxi University of Technology Hanzhong Shaanxi 723001 China; ^5^ Aerospace and Mechanical Engineering Department University of Notre Dame Notre Dame IN 46556 USA

**Keywords:** curved surface circuit, form following manufacturing, printed circuit, 3D printing, structural electronics

## Abstract

With the continuous development of digital surface circuit manufacturing technology, the three‐dimensional (3D) printing strategy of structural electronics has shown an increasingly wide application prospect in the field of electronics, especially in its main subfields such as conformal antennas, sensors, and aerospace. However, there are still many technical challenges in manufacturing high‐precision circuits on complex curved surfaces and within structures. In view of that, this study summarizes the latest development trends and results in 3D printing technology for structural electronics, focusing on the materials, conformal manufacturing technology, printing process control strategies, and their practical applications. In addition, this study analyzes the characteristics, advantages, and limitations of different digital surface circuit manufacturing processes and discusses their application potentials in practice. Finally, this study presents possible future development directions in the field of structural electronics 3D printing and emphasizes the opportunities and challenges in manufacturing conformal electronics on arbitrary surfaces.

## Graphical abstract

Conventional PCB etching (multi‐step) and indirect transfer techniques fail to meet structural electronics’ demands for lightweight, miniaturized, and customized manufacturing. 3D‐printed conformal circuits enable direct integration of conductive traces into substrates or onto surfaces, achieving integrated structural‐functional fabrication.

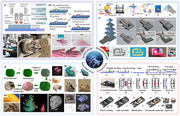

## 1 Introduction

In the digital era, miniaturization, light weights, and personalized customization requirements for electronic products have become the main development trends, posing technical challenges for future design and manufacturing. With the increase in circuit integration, electronic circuit manufacturing technology has evolved from traditional printed circuit boards (PCBs), as well as transistors, integrated circuits, and micro‐nano lithography, to 3D‐printed structural electronics. The phrase “structural electronics” refers to the 3D printing of circuit structures that are directly embedded within a three‐dimensional substrate or the construction of a conformal conductive layer on its surface to achieve the integration of electronic functions and physical structure integration. This technology has the advantages of reducing weight and process complexity, lowering costs, improving space utilization and layout flexibility, and supporting substrate shifts from planar to curved surfaces.^[^ ^1^, ^2^, ^3^, ^4^ ^]^

The creation of a conformal layout for circuits on curved surfaces breaks through the physical limitations of traditional planar PCB circuits, enabling the integration of multiple electronic components and circuit harnesses into a single 3D‐printed electronic product.^[^ ^5^ ^]^ This innovation offers unique advantages that are not possible with traditional planar electronics manufacturing, and it is particularly suitable for application scenarios in which complex geometries need to be accommodated. Examples include smart skins for the aerospace industry, bone repair scaffolds for medical implants, curved photovoltaic energy storage in the energy sector, and sensing networks inside robots.^[^ ^6^, ^7^, ^8^, ^9^ ^]^

Manufacturing technology of curved surface circuits can be roughly divided into indirect manufacturing and direct manufacturing.^[^ ^10^ ^]^ Indirect manufacturing transfers planar electronic devices to curved substrates using flexible substrates or bends them using external stimuli (e.g., heat, light, or magnetic stimuli). Transfer printing has been widely used due to its advantages of a large area preparation and a low cost.^[^ ^11^ ^]^ However, on complex hard surfaces, it is difficult for the indirect transfer process to realize complete conformal coverage;^[^ ^12^, ^13^ ^]^ also, ultra‐thin electronic structures are prone to interface fracture or unstable signals under conditions of strong bending or stretching.^[^ ^10^, ^14^, ^15^ ^]^ Therefore, how to achieve stable conformal coverage on complex hard surfaces while avoiding folds and buckles has become a major challenge in the manufacturing of curved circuits. The emergence of direct manufacturing technology has provided new ideas to solve this problem. By directly depositing the functional conductive material on an object's surface in a patterned manner or integrating the functional conductive material into an object, a stable conformal circuit and a curved surface structure can be realized, thus effectively improving the reliability and performance of the curved surface circuit.^[^ ^16^, ^17^ ^]^

**Figure 1:**
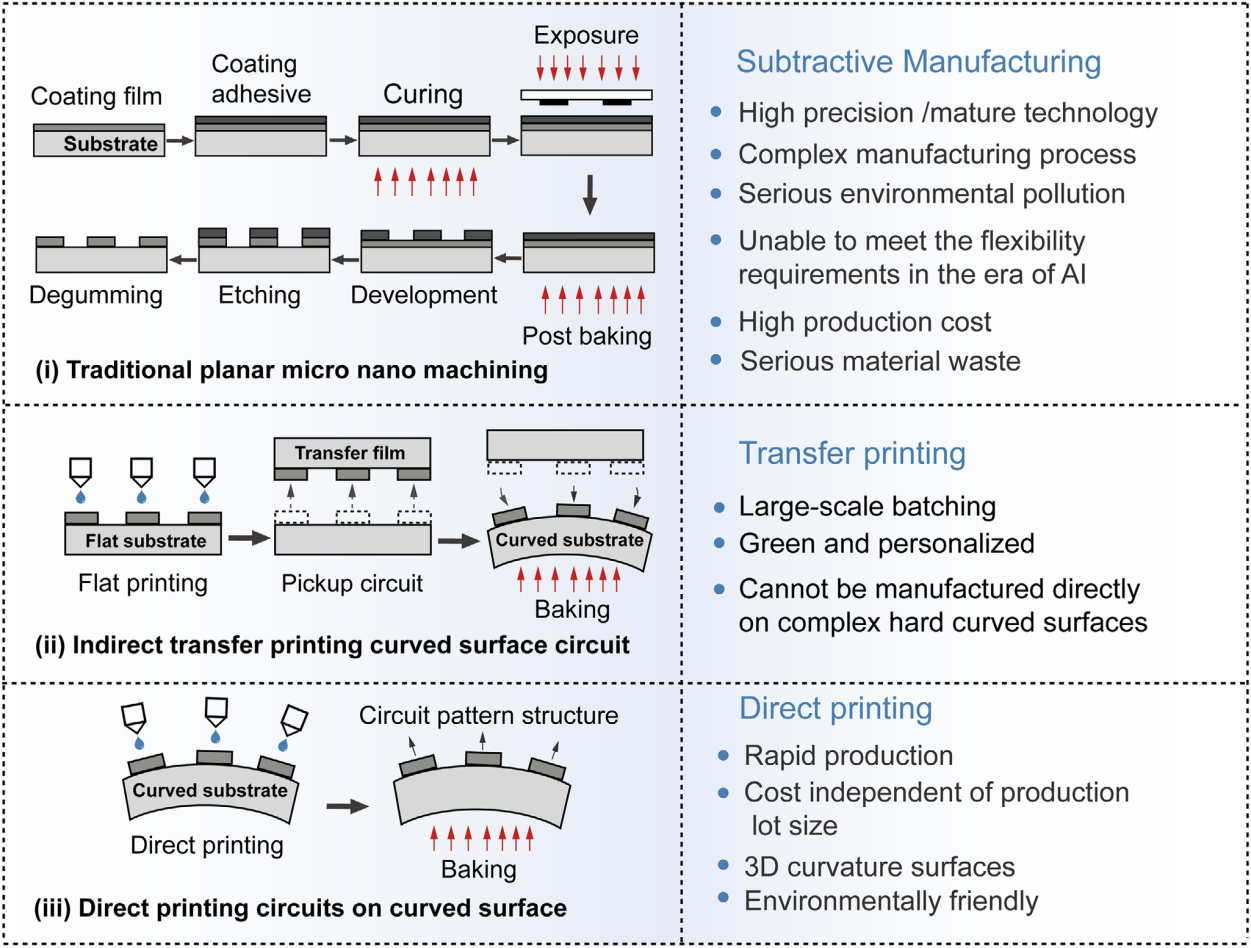
Difference between conventional planar micro/nanofabrication and curved circuit print fabrication i) Diagram of the conventional planar etched circuit fabrication process; ii) Indirect fabrication process of curved circuits based on flexible transfer printing; iii) Direct print fabrication process of curved circuits.

Currently, direct printing technologies for curved circuits mainly include direct‐write extrusion, inkjet printing, and laser direct‐write. Among these technologies, direct‐write extrusion is an area that has attracted great attention due to its simple process, high manufacturing efficiency, green environmental benefits, design flexibility, and low prototyping and production costs.^[^ ^18^, ^19^, ^20^ ^]^ A comparison of direct‐write extrusion for curved circuits with several other direct‐printing technologies is shown in **Table** **1**. The reason why 3D printing technology has received extensive attention in circuit manufacturing in recent years is due to the breakthrough progress in the fields of inorganic nanomaterials and printing electronics technology. **Figure** **1** illustrates the differences between traditional planar micro‐fabrication and nano‐fabrication, as well as printing processes for the circuit fabrication process.

**Table 1:** Comparison of direct manufacturing processes for curved circuits.

Technology	Type of material jetting			Material extrusion	Directed energy deposition
	Piezoelectric ink‐jet printing	Aerosol ejection	Electrohydrodynamic jetting	Direct writing extrusion	Laser direct writing
**Printing method**	On‐demand piezoelectric injection	Continuous flow	On‐demand and continuous injection	Microflow extrusion	Laser projection
**Working mechanism**	Inverse piezoelectric effect	Aerodynamics (atomization)	Electrical fluid dynamics	Layer‐by‐layer overlapping	Laser heat processing
**Resolution (µm)**	> 20	> 10	> 0.3	> 100	> 100
**Solution viscosity (cps)**	< 150	< 1000	< 10 000	< 500 000	—
**Deposition rate (mm s^−1^)**	≤ 400	≤ 200	≤ 100	< 50	> 200
**Material waste**	No	Yes	No	No	Yes
**Contact manner**	Non‐contact	Non‐contact	Non‐contact	Contact	Non‐contact
**Material compatibility**	Conductive ink, paint, graphene, and other nanocomposites	Atomizable solutions, such as metal inks, carbon nanotubes, graphene, and other nanocomposites	Conductive polymers, conductive inks, compounds	Liquid metal or metal paste, conductive carbon paste	Metals, polymers, composite materials
**Deposition shape**	Droplets	Slender strips	Droplets, mist, filaments	Slender strips	Lines
**Advantages**	High degree of digitization, good uniformity and consistency, as well as mature technology, material savings, and high efficiency	Wide range of material selection, high resolution	High resolution, high viscosity, and material printing	Nozzle not easily clogged, wide range of viscosities, simple and economical.	Low cost, high precision, and good flexibility
**Defect**	Nozzle can easily plug up, coffee ring effect, low resolution, low‐viscosity materials needed, and spray printing	No on‐demand injection, limited to customizable materials, solvent volatilization affected by the film‐forming effect and adhesion, and edge accuracy difficult to control	Non‐friendly to the printing of insulating materials, non‐uniform surface electric field, unstable printing	Low printing speed, low resolution, rough surface, and poor uniformity	Limited number of material and substrate types, low efficiency, narrow range, and small output
**Substrate geometry**	Moderate to highly curved surfaces	Mild to moderately curved surfaces	Mild to moderately curved surfaces	Mild to moderately curved surfaces	Planar and moderately curved surfaces
**Cost**	Medium	High	Higher	Low	Highest
**Manufacturable area**	Large	Large	Large	Large	Small
**Conductivity best‐case** **conductivity (S m^−1^)**	1.4 × 10^5^ Three‐dimensional printing of soft hydrogel Electronics	3.16 × 10^7^	2.5 × 10^6^	DW:1.56 × 10^5^ Metal injection: 2.30 × 10^6^ Wire embedding: 5.80 × 10^7^	2.2 × 10^6^
**Process complexity**	Simple	Complex	Complex	Simple	Simple
**Device application**	Curved surface circuits, antennas	Interconnections, conformal antennas, conformal sensors, sensor circuits Health monitoring systems 3D sensor structure conformal sensors	Electrode structures, RGO transistors, high‐resolution masking, metal network electrodes, transparent electrodes, sensors	3D polymer objects, microstrip antennas, sensors, microfluidic devices, and RF electronics	Curved PCB, RFID
**Trend**	High‐resolution and efficient manufacturing	High‐resolution and efficient manufacturing	High‐resolution and efficient manufacturing	High‐resolution and efficient manufacturing	Expanding applicable range
**Nozzle diameters (µm)**	10–50	100–300	0.3–30	300–800	‐
**Drop size (µm)**	25–200	2–5	Changes according to the nozzle diameter	350–800	‐
**Working distance (mm)**	0.25–1	1–5	< 1	< 0.1	<0.2
**Drop volume**	1–250 pL	1–5 fL	0.1 fL to several tens of pL	1–55 µL	‐
**Stability**	Medium (droplet‐dependent control)	Medium‐high (airflow aggregation)	Low‐medium (electric field sensitivity)	Medium‐high (stacking layer by layer)	High (precise energy control)
**References**	[272, 273, 274]	[4, 275]	[276]	[277, 278, 279, 280, 281]	[282, 283, 284, 285]

Traditional PCB manufacturing technology utilizes the planarization of the “motherboard” integration of discrete electronic components, and relies on external mechanical structures (such as frames and chassis) to achieve electronic system assembly. From the perspectives of process complexity and functional circuit patterning manufacturing, the traditional photolithography process needs to go through eight processes (such as mask preparation, etching, and plating). Flexible transfer technology is reduced to three steps (planar circuit preparation, surface transfer, and sintering), while a surface with direct printing technology requires only two steps (surface deposition and sintering), significantly simplifying the process and reducing manufacturing costs. This comparison shows that direct printing technology is simpler, more efficient, and cheaper. Electronics manufacturing technology is experiencing a paradigm evolution from “flat integration” to “curved surface with the shape of the manufacturing.” Based on an additive manufacturing strategy, direct sensing, conductive, insulating, and other multi‐functional units can be embedded in a three‐dimensional carrier structure to achieve the structure of functional circuits in design‐integrated manufacturing.

This study summarizes the latest research progress in 3D printing surface circuit technology globally, including materials, processes, applications, development prospects, and challenges. By analyzing the advantages and disadvantages of different materials and technologies, this study emphasizes the potential value of these technologies in practical application and discusses the opportunities and challenges introduced by the advent of 3D printing surface circuit technology.

## 2 Printing Manufacturing Process of Curved Surface Circuits

**Figure 2:**
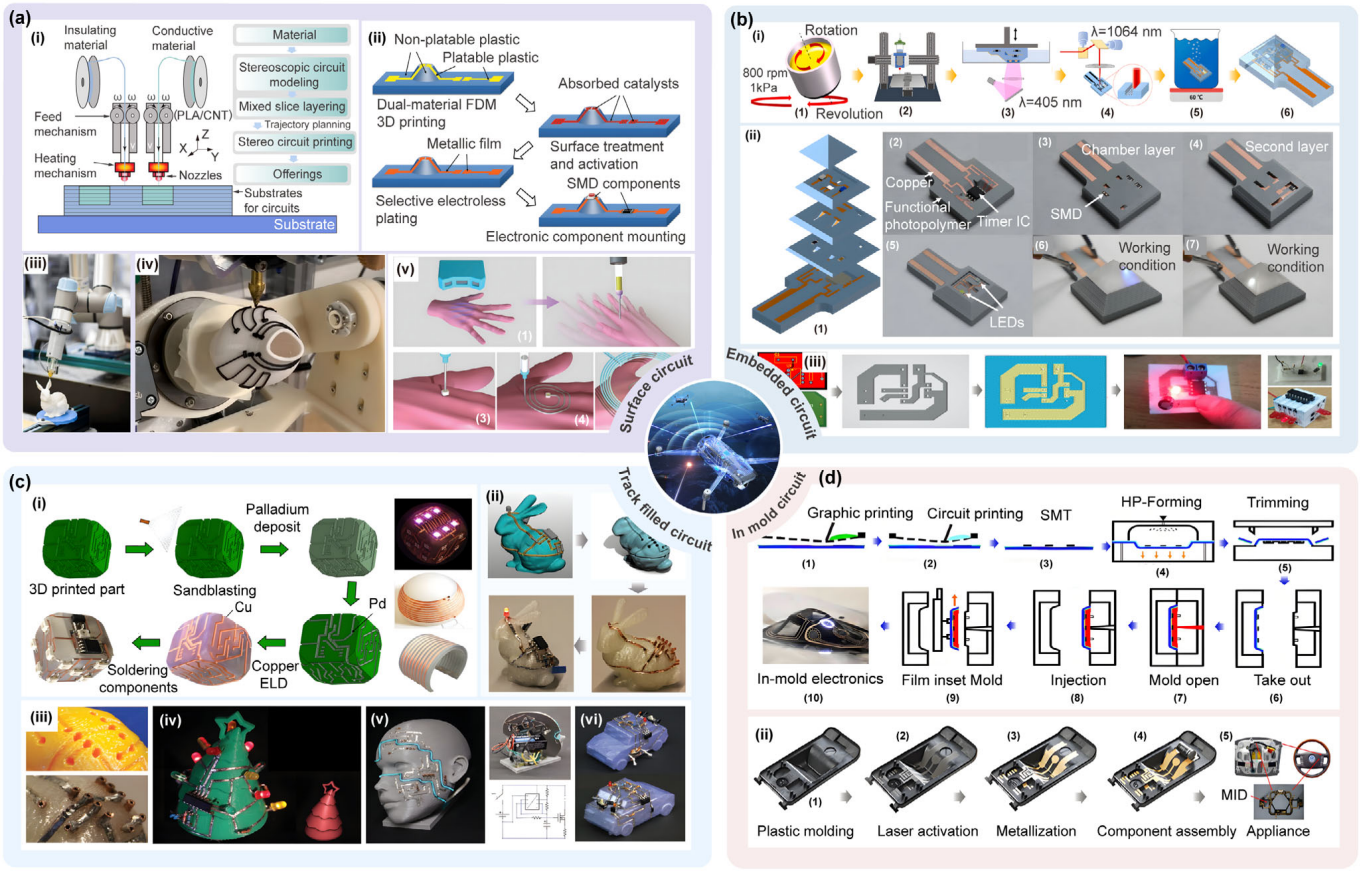
Comparison of printing processes for 3D printing surface circuits. a) Direct printing of surface circuits. i) Schematic of dual nozzle printing of conductive and insulating materials. ii) Schematic of the fabrication of selectively plated freeform circuits on 3D printed plastic structures. Reproduced with permission.^[^ ^23^ ^]^ Copyright 2022, Elsevier Science. iii) Direct printing of circuit structures on curved surfaces using a multi‐axis robotic arm. Reproduced with permission.^[^ ^24^ ^]^ Copyright 2020, Association for Computing Machinery. iv) Five‐axis multi‐material 3D printing of surface‐following circuits v) 3D printing of circuit structures on a moving freeform surface. Reproduced with permission.^[^ ^25^ ^]^ Copyright 2019, Wiley‐VCH. b) Embedded printed and solderless stereo circuits i) Hybrid strategy combining photopolymerization and laser activation to fabricate embedded 3D multilayer electronics. Reproduced with permission.^[^ ^26^ ^]^ Copyright 2023, Elsevier. ii) Schematic of embedded stereo circuit fabrication. Reproduced with permission.^[^ ^26^ ^]^ Copyright 2023, Elsevier. iii) Schematic diagram of the solderless stereo circuit fabrication process. c) Surface track‐filled printing. i) Dice surface circuits fabricated after 3D printing, metallization, and component soldering. Reproduced with permission.^[^ ^27^ ^]^ Copyright 2023, Elsevier. ii) Fabrication of curved surface circuits on a 3D structure. The electrical components are first positioned on the 3D shape and connected with bending traces. Next, channels for copper tape and holes for copper tubes are generated on the surface of the 3D structure. Finally, the tape is soldered to the electronic components to form a complete circuit structure. Reproduced with permission.^[^ ^28^ ^]^ Copyright 2017, IEEE. iii) (Top) Channels and holes in 3D printing and (bottom) the result after soldering. Reproduced with permission.^[^ ^28^ ^]^ Copyright 2017, IEEE. iv) Surface track circuit mounted on the surface of a Christmas tree. Reproduced with permission.^[^ ^28^ ^]^ Copyright 2017, IEEE. v) Example of a surface circuit mounted on a human face. Reproduced with permission.^[^ ^28^ ^]^ Copyright 2017, IEEE. vi) Example of a circuit mounted on the surface of an automobile. Reproduced with permission.^[^ ^28^ ^]^ Copyright 2017, IEEE. d) In‐mold electronic circuits. i) Schematic of the complete process flow for in‐mold circuit fabrication. Reproduced with permission.^[^ ^29^ ^]^ Copyright 2022, IOP Publishing. ii) Schematic of in‐mold electronics fabrication using laser patterning. Reproduced with permission.^[^ ^30^ ^]^ Copyright 2018, Springer.

The ideal manufacturing method for curved surface circuits is to construct a circuit by directly depositing conductive functional materials on the 3D curved surface in a continuous flow and extrusion manner using 3D printing technology. Currently, the 3D printing technology of curved surface circuits mainly includes two types of methods: Integrating the conductive circuit into an object and directly printing the circuit on an object's surface.^[^ ^21^ ^]^ For internally integrated processes, embedded solder printing, solderless stereo circuits, and embedded in‐mode circuits are typically used.^[^ ^22^ ^]^ Direct surface printing processes mainly include direct deposition and surface track filling printing. The current research status of the 3D printing surface circuits is illustrated in **Figure** **2**.

The surface direct printing of curved circuits involves the patterning of feature‐size circuits on complex curved substrates by extruding functional conductive materials layer‐by‐layer on a high‐precision multi‐axis motion platform. This printing process can achieve the structural redundant space of traditional processes and reduce the weight of electronic systems.^[^ ^31^ ^]^ Surface track‐filled printing involves the construction of conformal circuits by filling in prefabricated surface microfluidic structures utilizing conductive materials (e.g., silver nanopaste) as circuit tracks. Solder‐free three‐dimensional circuits form vertical interconnection channels between dielectric materials with the assistance of multi‐material co‐deposition technology. In conjunction with the pre‐set component insertion holes on the surface, these interconnection channels achieve the solder‐free conduction of printed circuits and components.^[^ ^32^ ^]^ Embedded circuit printing involves embedding circuits and components directly inside a 3D structural body. This type of circuit printing combines the processes of insulating substrate printing, component mounting, circuit printing, circuit and component soldering, and insulating material printing to achieve the integrated fabrication of fully functional circuit structures. In‐mold electronics (IME) technology, however, is used to print circuits on thermoformed substrates. This is achieved by using thermoforming and injection molding processes to fabricate flat substrates with the desired curved shapes.^[^ ^22^ ^]^ A comparison of the processes in terms of key metrics is shown in **Table** **2**.

**Table 2:** Process comparison of 3D printing surface circuits.^[^ ^24^, ^47^, ^48^, ^49^, ^50^ ^]^

Craft	Surface circuit direct printing	Track‐filled printing	Solderless stereo printing	Embedded circuit printing	In‐mode electronic circuit
**Resolution (µm)**	50–200	50–200	50–200	100–200	50–100
**Conductivity** **of electricity (Ω/^2^)**	Conductive ink/Conductive PLA, resistance value: 10^4^–10^6^	Conductive PLA/polymer‐metal composite, resistance value: 10^4^–10^6^	As low as 10^6^ depending on the conductivity of the filler material	Wires are embedded in the circuit and the resistance can be as low as 10^3^	High conductivity materials, such as copper foil and wire, are used, and the resistance can be as low as 10^2^
**Material**	Conductive PLA, conductive ABS, silver ink, copper powder, nickel powder, and other materials	Conductive PLA, silver ink, copper powder, nano copper wire, silver wire, and other materials	Conductive PLA, silver ink, copper powder, carbon black, and other materials	Copper wire, metal solder, conductive resin, silver ink, and other materials	Copper foil, thin film circuit, polymer‐metal composite material, silver ink, and other materials
**Process difficulty**	Simple, and suitable for prototype development and rapid iteration	Medium, with a complex track layout and high circuit connection requirements	High, with precise control of the printing process and use of the supporting structure	High, with high requirements for embedded circuits and welding accuracy	High, and involves in‐die metal forming and electronic packaging, necessitating precise control
**Circuit reliability**	Moderate. The surface connection might be damaged or affected by pressure	High. The filled track generally maintains better structural stability	Low. The hollow areas or uneven distribution of conductive material may cause circuit failure	High. The embedding can ensure that the circuit is securely connected.	High. The in‐mode electronic circuit has high reliability and durability
**Application scenario**	Small electronics, prototype development, wearable devices, and other applications	Equipment requiring complex circuit connections such as intelligent sensors	Smaller embedded electronic systems and prototyping	Complex circuit integration and embedded equipment with high power requirements	High‐performance and durable electronic products such as automotive electronics, and medical equipment

The surface direct printing of curved circuits involves patterning feature‐size circuits on complex curved substrates by extruding functional conductive materials layer by layer on a high‐precision multi‐axis motion platform, which can achieve the structural redundant space of traditional processes and reduce the weight of electronic systems.^[^ ^31^ ^]^ Surface track‐filled printing involves the construction of conformal circuits by filling in prefabricated surface microfluidic structures with conductive materials (e.g., silver nanopaste) as circuit tracks. Solder‐free three‐dimensional circuits form vertical interconnection channels between dielectric materials with the help of multi‐material co‐deposition technology, which, along with the pre‐set component insertion holes on the surface, achieves the solder‐free conduction of printed circuits and components.^[^ ^32^ ^]^ Embedded circuit printing involves embedding circuits and components directly inside a 3D structural body, combining the processes of insulating substrate printing, component mounting, circuit printing, circuit and component soldering, and insulating material printing to achieve the integrated fabrication of fully functional circuit structures. In‐mold electronics (IME) technology, however, is used to print circuits on thermoformed substrates, which is achieved by thermoforming and injection molding processes to fabricate flat substrates into the desired curved shapes.^[^ ^22^ ^]^ A comparison of the processes in terms of key metrics is shown in Table 2.

From design to manufacture, the 3D printing process of a curved circuit mainly includes four stages: Planar circuit design, curved circuit modeling, the direct printing of the curved circuit, and the post‐processing of the printed circuit. First, the schematic diagram of the planar circuit is designed according to the working principle of the functional circuit, and the PCB layout is carried out according to the standard. Then, according to the 3D matrix structure, the circuit layout and the component placement planning of the curved space are carried out. Subsequently, the CAM path process planning of the curved structure circuit is carried out, and the curved structure circuit is quickly and directly printed using 3D printing equipment along the generated processing path. The circuit, after printing, does not yet have electrical conductivity, so it is necessary to evaporate the ink solvent through thermal sintering treatment so that the printing line has good electrical conductivity. Finally, through functional testing and electrical conductivity characterization, the electrical performance of the circuit is verified, and the complete three‐dimensional surface circuit is manufactured.

### 2.1 Materials

**Figure 3:**
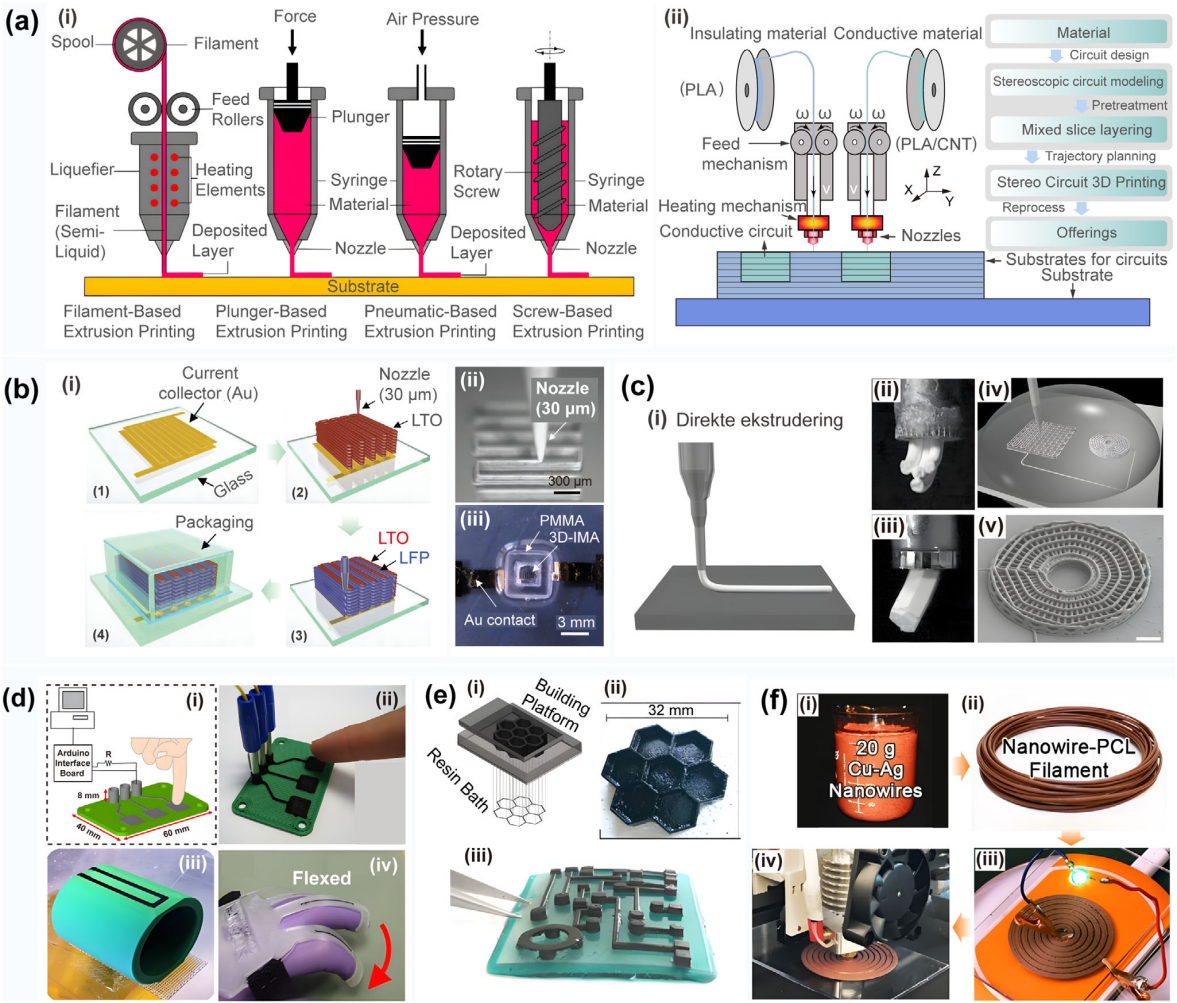
a) Overview of the traditional 3D printing process. Reproduced with permission.^[^ ^21^ ^]^ Copyright 2022, Elsevier Ltd: i) Several extrusion modes based on 3D printing; ii) Multi‐material 3D printing process. b) Multi‐layer circular structure for direct printing. Reproduced with permission.^[^ ^36^ ^]^ Copyright 2013, Wiley‐VCH: i) The schematic excitation of 3D interactive microwave architectures (3D‐IMA) fabricated on: 1) A gold current collector via printing; 2) Li_4_ Ti_5_ O_12_ (LTO); and 3) LiFePO_4_ (LFP) links through 30 µm nuzzles, followed by sintering, and 4) The packaging process; ii) An optical image of the LFP ink (60 wt% solids) position utilizing 30 µm noise to yield multiple structures; iii) An optical image of the 3D‐IMA composition of the LTO‐LFP electrodes after packing; c) Direct ink writing of 3D functional materials. Reproduced with permission.^[^ ^35^ ^]^ Copyright 2006, Wiley. i) Schematic illustration of direct ink writing techniques; ii) An optical image of a photoresist, electrocast, and molded (LIGA) gear‐shaped nozzle tip, and a corresponding optical image of the ink filament formed by the extrusion thereof; iii) The UV‐LIGA μ‐tip with a 560 µm vertex‐to‐vertex hexagonal orifice and the corresponding optical image of the ink filament formed by the extrusion thereof; iv) A schematic of the process of direct ink position on a curved surface. A syringe containing concentrated polyelectrolyte ink is immersed in a solidification cell (gray hemispherical droplets) and then deposited onto a glass substrate; v) Optical images acquired in situ during the deposition process showing the actual features presented in (c)‐(iv), including the deposition nozzle that is patterning the 3D periodic lattice, and an image of the 3D radial array completed alongside this structure; vi) The 3D periodic lattice with simple tetragonal geometry consisting of eight layers with a bar diameter of 1 µm; d) The direct 3D printing strategy of the electronic sensors used for printing a capacitive interface device. Reproduced with permission.^[^ ^40^ ^]^ Copyright 2012, PLOS ONE: i) The CAD design of the printed interface device, and a simple circuit used to detect inputs; ii) A photo of the printed device; iii) 3D printing of a capacitive “smart” vessel. The vessel during the printing process shows the embedded sensor strip; (iv) 3D printing of the flex sensors; **(e)** The 3D printing strategy with the CNT for enhanced electrical performance. Reproduced with permission.^[^ ^46^ ^]^ Copyright 2017, Elsevier: i) The representative scheme of the DLP 3D printing process; ii) 3D hexagonal structure with a thickness of 5 mm containing 0.1 wt.% CNTs; iii) A circuit‐like structure built on an insulating base with suspended elements containing 0.1 wt.% CNTs; f) The synthesis of multiple copper‐silver core‐shell nanowires used in 3D printed electronics. Reproduced with permission.^[^ ^47^ ^]^ Copyright 2018, Wiley: i) Synthesis of composite nanowire raw materials; ii) Generation of high‐conductivity polymer filaments; iii) Direct extrusion printing of composite conductive filaments; iv) Illustration of Cu–Ag NW filament 3D printing of inductive charging coils for wireless power supply of LEDs.

In traditional 3D printing, a single‐nozzle printing circuit faces certain limitations in terms of raw material types and electrical properties, which impacts the performance of 3D printing electronic products.^[^ ^33^, ^34^ ^]^ Multi‐material printing eliminates the printing limitations of single material properties and enables 3D printing products to be applied in more fields. Multi‐material printing mainly involves utilizing additional nozzles to deposit new materials (i.e., polymer substrates and conductive paths). By co‐printing different types of materials, such as conductors, semiconductors, and dielectrics, with multiple nozzles, multi‐functional integration can be realized in 3D printing devices, which can significantly improve the overall printing performance,^[^ ^23^, ^35^ ^]^ as shown in **Figure** **3**a. For instance, K. Alhassoon^[^ ^36^ ^]^ successfully fabricated lithium‐ion microcells with high energy density and power density using multi‐material printing, as displayed in Figure 3b. G.L. Goh^[^ ^37^ ^]^ proposed an effective strategy to design and fabricate integrated multi‐functional structures using soft, rigid, and conductive materials (such as polymers, polymer nanocomposites, and metals) in multi‐material 3D printing. The embedded metal wire, manufactured using the internal wire embedding technology, forms a reliable high‐current load interconnection for the conductive joint acting as the heater. In 3D printing of curved surface circuits, a common conductive material is made of composite plastics doped with conductive components, as presented in Figure 3c. These composites typically consist of a polymer matrix containing conductive nanofillers, such as graphene,^[^ ^38^ ^]^ carbon nanotubes,^[^ ^39^ ^]^ carbon black,^[^ ^40^ ^]^ and metal particles,^[^ ^41^ ^]^ and have been widely used in manufacturing of circuit and electrical components; typical applications include multi‐layer circuit boards,^[^ ^34^ ^]^ electrical connectors,^[^ ^42^ ^]^ sensors,^[^ ^43^ ^]^ and antennas.^[^ ^41^, ^44^ ^]^

Currently, conductive composite wire materials suitable for fusion deposition modeling (FDM) printers available in the market include carbon fiber/PLA composite materials, graphene/PLA composite materials, carbon black/ABS composite materials, and carbon fiber/PETG composite materials. The performance comparison of these materials is given in **Table** **3**. It should be noted that a variety of electrical functions can be achieved by incorporating different types of conductive fillers in thermoplastics.^[^ ^45^, ^46^ ^]^ For instance, S.J. Leigh^[^ ^40^ ^]^ introduced a conductive thermoplastic composite formulation that could successfully print out sensors for sensing mechanical bending and capacitance changes, as shown in Figure 3d. G. Gonzalez^[^ ^46^ ^]^ optimized the viscosity and dispersion stability of a 3D printing circuit by adding a reactive diluent to the acrylic acid formulation, thus improving its conductivity, as shown in Figure 3e. Further, M. A. Cruz^[^ ^47^ ^]^ combined nanowires with a carbon‐based hybrid material to prepare a 3D circuit structure with excellent conductivity, which is displayed in Figure 3f. In addition, Cruz reported on a silver‐coated copper nanowire with a resistivity of 0.002 Ω cm, which could be successfully used for circuit preparation. In addition, X. Wei^[^ ^48^ ^]^ developed conductive filaments with a resistivity of 0.006 cm for low‐cost fuse manufacturing (FFF) 3D printing, which could be used to print microstrip transmission lines, super‐surface antennas, and 3D metamaterials.^[^ ^49^, ^50^ ^]^ K. Alhas^[^ ^51^ ^]^ successfully designed a feed loop antenna with a gain of 5–8 dBi by extracting the complex dielectric constant of 3D printed filaments through fitting scattering parameters. In addition, I. E. Stewart^[^ ^52^ ^]^ fabricated excellent 3D circuits using an electrified composite filled with metal particles.

**Table 3:** Performance comparison of the FDM conductive composites.^[^ ^23^, ^286^, ^287^, ^288^, ^289^, ^290^, ^291^, ^292^, ^293^ ^]^

Material type	Conductivity (S m^−1^)	Mechanical strength (Mpa)	Melting point (°C)	Thermal stability (°C)	Chemical resistance	Volatility (g m^−3^)	Viscosity (Pa s)	Application occasion
**Conductive PLA/carbon fiber reinforced polymer**	10^3^–10^5^	50–70	180–230	100–200	Ordinary acid and alkali resistance and poor solvent resistance	Low	0.1–0.5	Suitable for low conductivity requirements, basic electronics applications
**Conductive PLA/graphene composite**	10^5^–10^6^	70–100	180–230	150–250	Good chemical resistance and excellent acid and alkali resistance	Very low	0.08–0.4	Suitable for high conductivity requirements, electronic components
**Conductive ABS/carbon black composite**	10^2^–10^4^	60–90	220–240	150–250	Solvent, and UV and oxidation resistant	Medium	0.2–0.7	For applications requiring high mechanical strength
**Conductive PETG/carbon fiber reinforced polymer**	10^3^–10^4^	70–100	220–250	180–270	Corrosion and abrasion resistance, and poor resistance to solvents	Low to Medium	0.3–0.6	Conductive parts suitable for high strength requirements
**Conductive nylon/metal powder composite**	10^3^–10^5^	80–120	220–260	180–250	Good chemical resistance, acid and alkali resistance, and solvent resistance	Low	0.2–0.7	High strength conductive parts suitable for complex shapes
**Conductive TPU/carbon black composite**	10–10^3^	20–40	230–260	200– 260	Good oil and water resistance, but not resistant to high temperatures	Medium	0.1–0.5	Suitable for flexible components, and commonly used in soft electronics applications

Currently, material and process optimization represents a key direction in the research of the electrical performance of 3D printing circuits. From a material point of view, although advanced composite conductive wires, such as electrified and silver‐coated copper nanowires, have high conductivity, their resistivity is significantly higher than that of bulk copper of 1.67 μ Ω cm. For instance, electrified and silver‐coated copper nanowires have 3750 and 1250 times higher resistivity than block copper, respectively. This is primarily due to the differences in conductivity for composites, particularly the embedding of metal particles in thermoplastic matrices, which can easily result in poor electrical contact.^[^ ^47^ ^]^ Furthermore, the conductivity of composites is very sensitive to temperature changes. At high temperatures, the conductivity can significantly decrease and may even be one order of magnitude lower than the conductivity at room temperature.^[^ ^42^ ^]^ This poses great challenges to the high‐temperature melt extrusion process.^[^ ^53^ ^]^

In addition to material optimization, process optimization is also crucial for achieving good performance. The manufacturing quality of 3D printing circuits directly defines the final electrical property. For instance, the interlayer bonding strength, current transmission path design, and printing parameters, such as temperature, speed, and filling density, during printing directly affect the conductivity and thermoelectric characteristics of the printed circuit. To solve the conductivity problem of materials, researchers have made many innovations in the printing process, attempting to improve the electrical contact, reduce the insulation effect of thermoplastic by optimizing the printing parameters, and improve the conductivity of composite materials by employing post‐treatment technologies, such as heat and chemical treatments.

The material properties of substrates have an important influence on the electrical properties of 3D‐structure electronic products.^[^ ^54^, ^55^ ^]^ Surface cleanliness, surface wettability, adhesion, porosity, and surface roughness are the key factors that affect performance.^[^ ^56^ ^]^ When the surface cleanliness of a substrate is poor, the wettability of the ink will be reduced. The dust particles in the deposited ink may lead to an insulation effect, thereby reducing the electrical properties of the printed electronic components.^[^ ^57^ ^]^ The surface energy of a substrate reflects the ability of solid surface molecules to adsorb and allow fluid adhesion.^[^ ^56^ ^]^ High surface energy substrates generally have better wettability and adhesion.^[^ ^58^ ^]^ For low surface energy substrates, the surface energy can be improved using surface methods such as plasma treatment or laser treatment, thereby enhancing the adhesion of ink. Surface tension is the cohesive force between liquid molecules, and it affects the printability and wettability of ink on the substrate surface. The surface tension of the ink must be lower than the surface energy of the substrate to ensure good wettability.^[^ ^59^ ^]^ If the surface tension of the ink is high, the ink will bead up on the surface of the substrate, resulting in discontinuous lines or printing defects. Surface wettability is the ability of a fluid to diffuse on the surface,^[^ ^60^ ^]^ which is usually measured using the fluid contact angle. Good surface wettability helps to improve print resolution and feature consistency. Plasma treatment can improve the surface wettability of polymer substrates with chemical and physical methods. Adhesion is also affected by the surface energy between the ink and the substrate. Common technologies used to enhance ink adhesion include plasma treatment, chemical and flame treatment, surface roughening, and the addition of adhesion promoters to ink. The porosity of a substrate is determined by the size and number of pores, which directly affects permeability and may lead to ink penetration or absorption into the substrate. Studies have shown that highly permeable substrates often lead to lower electrical conductivity. The surface roughness of a substrate has a negative effect on ink deposition and printing quality, which may lead to line breaks or point deletions.^[^ ^61^, ^62^ ^]^ This is especially true when the ink layer is thin because the surface valley cannot obtain enough ink filling.^[^ ^63^ ^]^ In addition, conductive inks such as silver nano‐inks or conductive silver pastes generally require thermal sintering during the printing process. To avoid the fracture of a line due to thermal expansion or deformation during the sintering process, the thermal decomposition temperature of the substrate material should be higher than the thermal sintering temperature of the conductive ink. For polymer substrates with poor temperature resistance (such as PET), the thermal stability can be improved with surface treatment (such as plasma treatment).

The most commonly used substrate materials include ceramics, glass, polymers (such as polycarbonate (PC), polyethylene naphthalate (PEN), polyethylene terephthalate (PET), and polyimide (PI)), metals, paper, and metal foils.^[^ ^56^ ^]^ Glass substrates are widely used in photovoltaics, displays, and lighting because of their excellent surface qualities, chemical resistance, thermomechanical stability, high optical transparency, and moisture and oxygen resistance.^[^ ^64^ ^]^ In contrast, the thermal stability of a polymer substrate is low, and the glass transition temperature is usually 150°C or lower, so a polymer substrate is not suitable for exposure to a high‐temperature environment during the sintering process. Otherwise, deformation or deterioration may occur. Therefore, it is very important to adopt selective and low‐temperature sintering technology for these types of substrates.^[^ ^56^ ^]^

Paper substrates have been used in a variety of innovative applications due to their flexibility and foldability. Example applications include foldable circuits^[^ ^65^ ^]^ and flexible displays.^[^ ^66^ ^]^ Metal foils are also widely used as substrates for printed electronic applications (such as top‐emitting organic light‐emitting diode (OLED) displays, OLED lighting,^[^ ^67^ ^]^ organic thin‐film transistors (OTFTs),^[^ ^68^ ^]^ TFT display backplanes, and organic photovoltaic cells). Metal foil has the advantages of low moisture permeability, dimensional stability, chemical resistance, high heat resistance, and flexibility. However, due to its electrical conductivity, it is not feasible to directly deposit functional inks on the surface of metal foils, and it is usually necessary to coat the surface with an electrically insulating passivation layer to prevent the short‐circuiting of printed patterns. The types of common substrate materials are shown in **Table** **4**.

**Table 4:** Comparison of common circuit structure substrate types.^[^ ^20^, ^21^, ^56^, ^167^, ^275^, ^294^, ^295^, ^296^, ^297^, ^298^ ^]^

Material type	Thermal stability/°C	Surface roughness/µm	Chemical properties	Surface flatness/µm	Electrical properties	Applicable situation
**Alumina ceramics**	> 1000	0.1–0.3 (After polishing)	Polar/inert	Extremely high	Insulativity	High‐temperature electronic components
**Polyimide (PI)**	155–270 (Glass transition)	0.3–0.5 (After treatment)	Polarity	High	Low dielectric loss	Flexible circuit, high‐frequency electronics
**Thermoplastic polyurethane (TPU)**	80–150 (Processing temperature)	0.2–0.4 (After laser treatment)	Polarity (after treatment)	Medium	Anisotropic electrical conduction	Wearable devices, anti‐icing system
**Polyether ketone ketone (PEKK)**	395 (Forming temperature)	< 0.1 (Carbon fiber composite)	Polarity	Extremely high	Insulativity	High‐strength structural electrons
**Glass substrate**	> 600 (Softening point)	0.05–0.1 (Polishing)	Polarity	Extremely high	Low dielectric loss	High‐frequency circuit, transparent electrode
**Metal** **(Stainless steel)**	> 800 (Melting point)	0.5–1.5 (After sandblasting)	Inertia	Medium	Conductivity	High‐power electronic heat dissipation substrate
**Metal foil** **(Copper foil)**	1085 (Melting point)	0.1–0.3 (Electrolytic polishing)	Inertia	High	Conductivity	High‐frequency circuit conductive layer
**Paper‐base**	< 100 (Temperature resistance limit)	10–50 (Native fiber)	Inertia	Low	Insulativity	Low‐cost disposable electronics

### 2.2 Direct Printing of Surface Circuit

**Figure 4:**
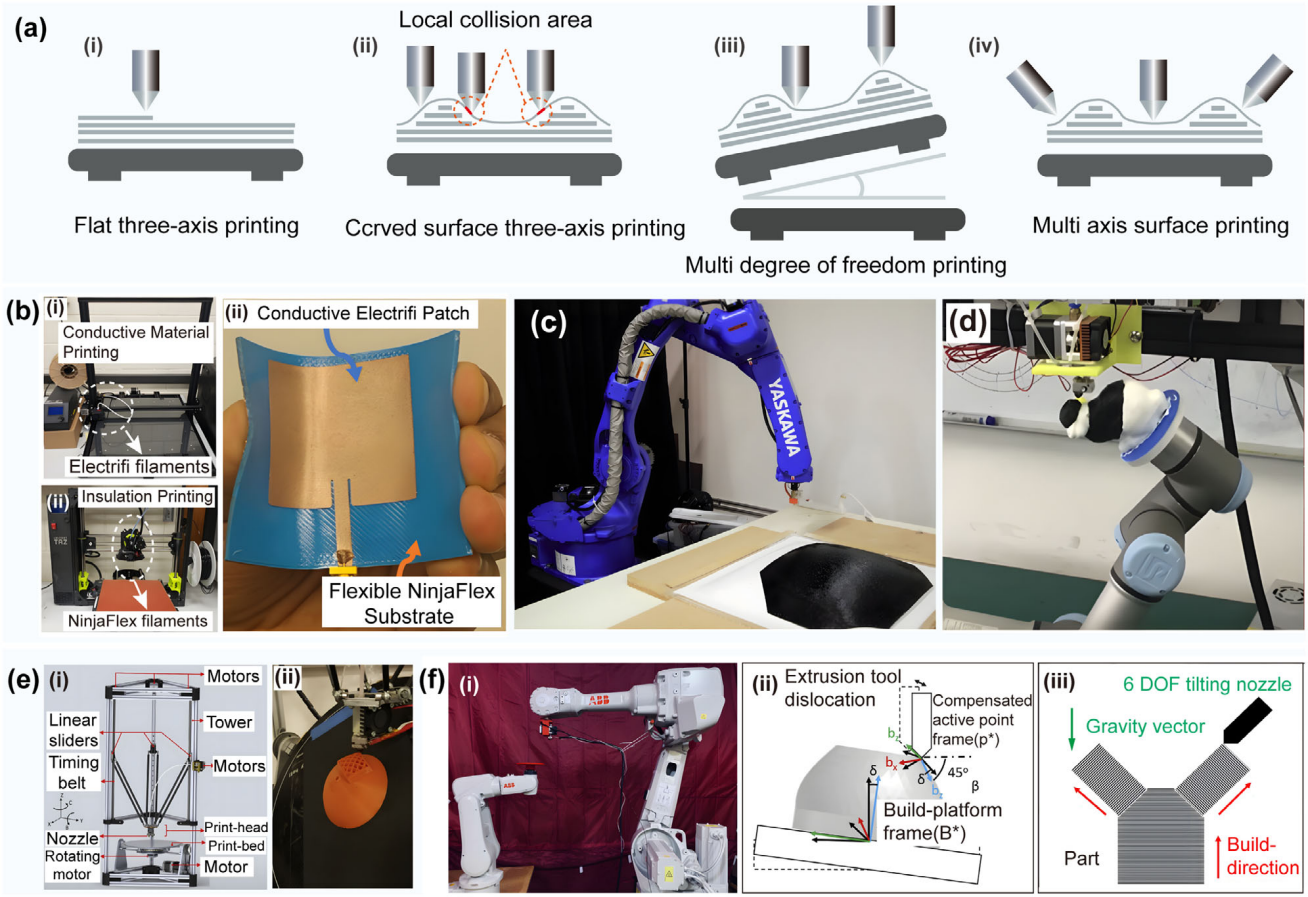
**a)** Analysis and contrast for three‐axis printing and multi‐degree‐of‐freedom printing. b) Direct printing of a flexible microstrip antenna based on conductive and non‐conductive NinjaFlex fibers. Reproduced with permission.^[^ ^69^ ^]^ Copyright 2021, MDPI AG: i) A 3D CR‐10 DIY high‐precision 3D printer for the printing of conductive wires; ii) A LulzBolt Taz6 3D printer for the printing of NinjaFlex substrates; iii) An electronically‐based micro‐strip antenna on a flexible NinjaFlex substrate for 3D printing; Multi‐degree‐of‐freedom 3D printing approach for non‐planar surfaces. c) The setup for generating a robot trajectory for conformal 3D printing using a non‐planar layer. Reproduced with permission.^[^ ^79^ ^]^ Copyright 2022, ASME. d) A 6‐DOF robot for changing the construction direction in intermittent steps during the production process. Reproduced with permission.^[^ ^88^ ^]^ Copyright 2017, IEEE. e) The five‐axis additive manufacturing of free‐form models by constructing transition layers. Reproduced with permission.^[^ ^83^ ^]^ Copyright 2019, ELSEVIER LTD. i) The axis 3D printer design showing system actuators and mechanical components; ii) Real‐time manufacturing using the designed five‐axis 3D printer, showing all the proposed paths of the transition layers. f) The fabricated curved circuit structures using unsupported extrusion rapid prototyping techniques. Reproduced with permission.^[^ ^84^ ^]^ Copyright 2020, Elsevier: i) Two manipulators used to fix the three‐degree‐of‐freedom (3‐DOF) build platform and the experimental setup of the 3‐DOF extrusion tool. ii) The process of changing the extrusion tool's position by reorienting the platform to avoid the support structure.

The process of directly printing a circuit on a curved surface differs from the traditional planar manufacturing process. Namely, traditional planar 3D printing relies on a three‐axis motion system consisting of *x*, *y*, and *z* axes, where materials are extruded and stacked layer by layer in the *z*‐axis direction to form a planar structure. Due to the constraints imposed by the degrees of freedom, three‐axis printing is susceptible to collisions with the print surface, as depicted in **Figure** **4**a. D. Mitra^[^ ^69^ ^]^ printed a patch antenna with good impedance matching and a 2.4 GHz resonant frequency on a cylindrical surface utilizing the FFF method with Electrifi filament as a conductive trace, as shown in Figure 4b. However, this approach faces significant challenges for oblique or curved 3D structures.^[^ ^70^ ^]^ Although these devices possess three degrees of freedom, the limitation that the nozzle can only deposit material along a single axis fundamentally places this approach in the category of 2.5D printing technology. Due to the limitation of the three‐axis printing system, the sprinkler head cannot always be perpendicular to a curved surface,^[^ ^71^ ^]^ making the material prone to deformation, discontinuity, and outflow on the inclined surface, which affects the conductivity and can lead to interlayer connection fracture, thus reducing both the accuracy and quality of the printing process.^[^ ^72^ ^]^ For example, C. Li^[^ ^73^ ^]^ employed a three‐axis printer to deposit conformal circuits onto micro‐surfaces. However, it was observed that the heightened curvatures led to circuit fractures and inconsistent morphologies, eventually giving rise to a “tadpole‐like” shape.

The enhancement of the print head and platform with additional degrees of freedom makes the execution of more flexible motion control strategies possible. The emergence of multi‐degree‐of‐freedom additive manufacturing systems has allowed 3D printing to surpass the limitations of traditional linear fabrication techniques. This advancement has further enabled the printing of complex segments and curves on parts with irregular shapes and reduced the anisotropy issues stemming from discontinuous material deposition. The diverse structures of prevalent multi‐degree‐of‐freedom 3D printers include parallel mechanisms,^[^ ^74^, ^75^ ^]^ rotary structures,^[^ ^76^, ^77^ ^]^ robotic arm configurations,^[^ ^78^, ^79^, ^80^, ^81^ ^]^ and hybrid actuation systems,^[^ ^82^, ^83^, ^84^, ^85^, ^86^ ^]^ as illustrated in Figure 4c,d.

The multi‐axis 3D printing method can be performed in two ways, by rotating either the printing bed or the extrusion head. For instance, in five‐axis printing, in addition to the three traditional axes, *x*, *y*, and *z*, the *b*‐axis rotating around the *y*‐axis and the *c*‐axis rotating around the *z*‐axis are also added. Through the coordination and linkage of each axis, the nozzle's orientation is adjusted so that the nozzle can always be perpendicular to the surface to be processed. X. Song^[^ ^74^ ^]^ developed an affordable six‐degree‐of‐freedom parallel 3D printing machine. Y. Ding^[^ ^85^ ^]^ engineered an eight‐degree‐of‐freedom 3D printing setup that consisted of an inclined platform and a six‐axis robotic arm. Y. Zhao^[^ ^87^ ^]^ proposed a five‐axis linkage printing control system, which was specially developed to control the precise deposition of conductive slurry on a complex curved surface. F. Hong^[^ ^82^ ^]^ transformed the traditional three‐axis printer into a five‐axis system, thus providing greater printing freedom by reducing the contact angle between the extrusion head and the PLA substrate. The five‐axis printing strategy proposed by M.A. Isa^[^ ^83^ ^]^ could effectively solve the problems related to the step effect and support in traditional 3D printing and promote the realization of curved surface paths, as displayed in Figure 4e.

In addition, the stentless extrusion technique proposed by P.M. Bhatt^[^ ^84^ ^]^ could also be used for the precise construction of curved thin shell parts, as shown in Figure 4f. J. A. Gardner^[^ ^89^ ^]^ found that when curved surface samples were printed using a five‐axis system, the five‐axis printing system performed better in terms of surface finish and structural strength than conventional three‐axis printing systems. Y. Shaw^[^ ^90^ ^]^ successfully printed circuit patterns on complex curved surfaces using MXene water‐based inks and pneumatic extrusion heads for five‐axis machines. By employing these innovative multi‐axis printing technologies, the limitation of traditional three‐axis printing on complex curved surfaces could be overcome, and significant breakthroughs in terms of precision, conductivity, and structural strength of circuit printing could be achieved.

**Figure 5:**
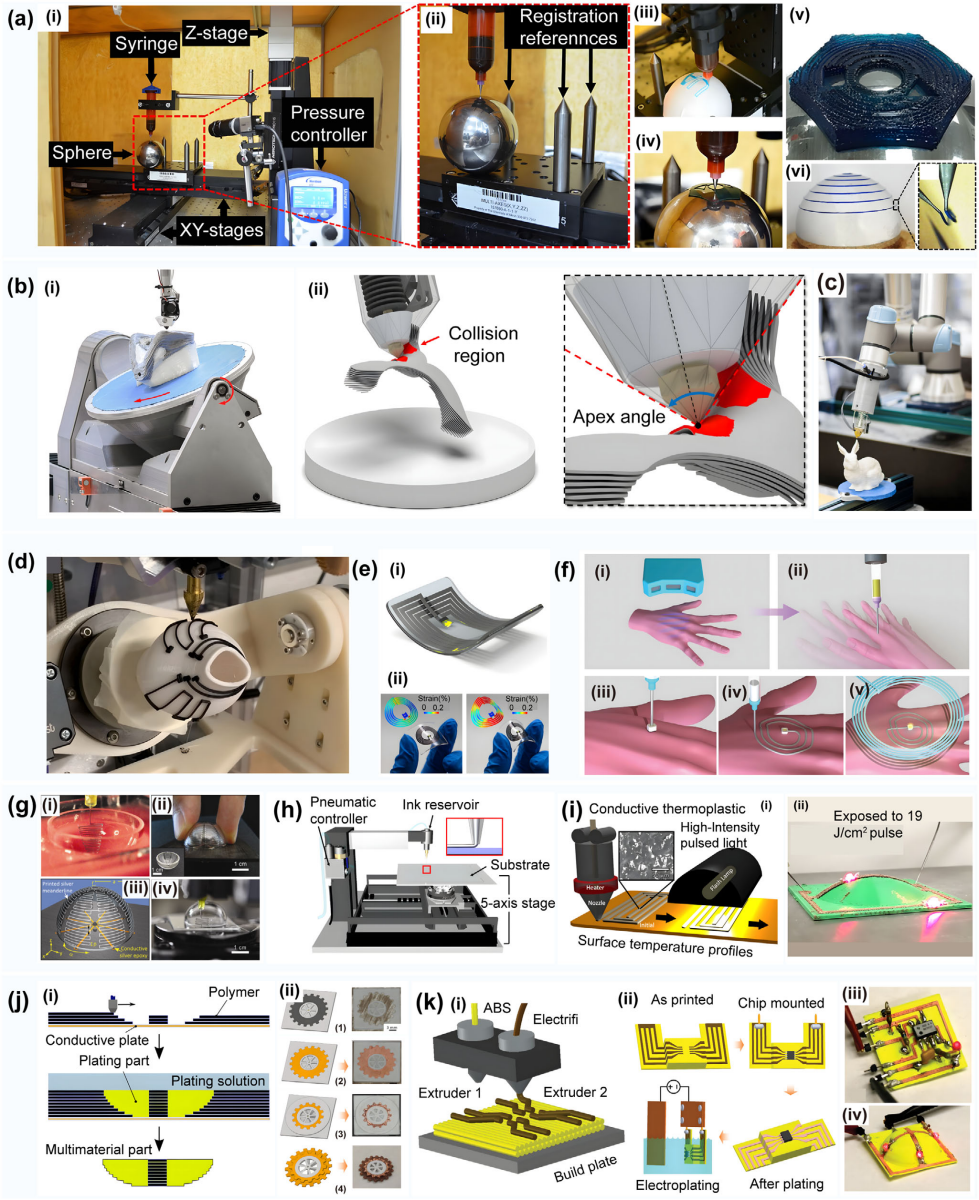
**a)** Conformal additive manufacturing using a direct‐print process. Reproduced with permission.^[^ ^92^ ^]^ Copyright 2019, Elsevier: i) The setup of the system components. ii) The setup used to determine the origin point on the sphere that is used to locate the points on the substrate's surface. 3D printing of a UA and hexagonal‐shaped models with internal features on a sphere‐shaped substrate using the fully conformal slicing method. iii) 3D printing of the first layer of the UA part. iv) 3D printing of the first layer of the hexagonal‐shaped part. v) The complete print of the hexagonal‐shaped part. vi) Direct printing of lines using different angles between the nozzle (in a vertical orientation) and the freeform substrate. b) Reinforced FDM: Multi‐axis filament alignment with controlled anisotropic strength. Reproduced with permission.^[^ ^24^ ^]^ Copyright 2020, Association for Computing Machinery. i) Tool paths optimized to supervise the fabrication of curved layers with a dual‐material multi‐axis 3D printer using the fused deposition of filaments; ii) Illustration of collision detection and the bounding volume of a specially designed printer head with a small apex angle. c) Multi‐axis 3D printing of curved layers achieved with hardware equipped with a UR5e robotic arm. Reproduced with permission.^[^ ^93^ ^]^ Copyright 2022, Association for Computing Machinery. d) Five‐axis multi‐material 3D printing of curved electrical traces. Reproduced with permission.^[^ ^9^ ^]^ Copyright 2023, Elsevier. e) Room‐temperature high‐precision printing of flexible wireless electronics based on MXene inks. Reproduced with permission.^[^ ^90^ ^]^ Copyright 2022, Nature Publishing Group. i) Fabrication and mechanism of printed MXene NFC tags that communicate wirelessly via a smartphone and capture the power to light up an LED. ii) Optical images of the curved small‐sized NFC tags and the corresponding strain distributions of the flexible antennas with bending (as shown in the inset). f) 3D printed functional and biological materials on moving freeform surfaces. Reproduced with permission.^[^ ^25^ ^]^ Copyright 2018, Wiley‐VCH. i) 3D scanning of the target surface that will be printed on; ii) Real‐time tracking of the rigid‐body motion of the target surface; iii) Schematic images of the pick‐and‐place process of discrete electronic components on the target surface using a vacuum nozzle; iv) Direct writing of conductive ink; v) Illustration of the powering up of the LED via a wireless power transmission system; g) Conformal printing of electrically small antennas on 3D surfaces. Reproduced with permission.^[^ ^94^ ^]^ Copyright 2011, Wiley‐VCH: i) An optical image of an antenna (ESA4) printed onto the interior surface of a glass substrate embedded in a PDMS mold; ii) Optical images of this antenna before (inset) and after connection to the feedlines; iii) Schematic illustration of an electrically small antenna with labeled geometric parameters; iv) Optical image of an antenna during the printing process. h) High‐resolution, reconfigurable printing of liquid metals with 3D structures. Reproduced with permission.^[^ ^95^ ^]^ Copyright 2019, American Association for the Advancement of Science: i) Flash ablation metallization of conductive thermoplastics;^[^ ^96^ ^]^ 1) Illustration of the FFF printing and pulsed light exposure process with the filament surface morphology highlighted before and after the exposure; 2) The pulsed‐light exposed 3D PCBs. j) Multi‐material additive manufacturing of polymers and metals using fused filament fabrication and electroforming. Reproduced with permission.^[^ ^97^ ^]^ Copyright 2019, Elsevier: i) A schematic diagram and real parts of the molding process introduced by Matsuzaki; ii) The fabricated gear shapes as well as the schematic diagram and photographs of the molding process. k) Selective electroplating for 3D‐printed electronics. Reproduced with permission.^[^ ^98^ ^]^ Copyright 2019, John Wiley & Sons: i) Dual extrusion fused filament fabrication; ii) Electroplating process; 3D printed unstable 555 timer circuit presented in (c) tested both iii) after soldering and iv) after the LEDs are turned on.

One of the major challenges in achieving high‐precision multi‐axis 3D printing is the generation of a slice system that adapts to the path of a curved surface.^[^ ^82^, ^91^ ^]^ To this end, researchers have developed a multi‐axis slice algorithm and visualization model software.^[^ ^24^, ^92^ ^]^ F. Alkadi^[^ ^92^ ^]^ studied the algorithm of surface conformal slicing and realized conformal printing using a three‐axis motion platform, as illustrated in **Figure** **5**a. G. Fan^[^ ^24^ ^]^ proposed an innovative surface calculation framework for the layered and tool path generation for multi‐axis 3D printing, which could enhance the model's strength by arranging filaments in the direction of higher stress, as shown in Figure 5b. T. Zhang^[^ ^93^ ^]^ developed a curved layer slicing algorithm for multi‐axis 3D printing and successfully realized direct printing of curved surface circuits on hardware equipped with a UR5e mechanical arm, as presented in Figure 5c.

Further, F. Hong^[^ ^9^ ^]^ optimized the curved surface slicing system and printed the conformal circuit, which is presented in Figure 5d, on the free‐form surface substrate using a multi‐material five‐axis 3D printer. This strategy could reduce the resistivity of the printing circuit by more than nine times and effectively eliminate the bleed and wire drawing problems. In addition, Y. Shao^[^ ^90^ ^]^ realized non‐contact layer‐by‐layer printing of high‐viscosity silver paste on a non‐planar surface by optimizing the curved surface slicing system, as displayed in Figure 5e, but the achieved printing accuracy was not satisfactory. Aiming to further improve the printing quality, Z. Zhu^[^ ^25^ ^]^ integrated the closed‐loop feedback control system into a 3D printer, thus significantly improving the printing accuracy of the curved surface circuit, as shown in Figure 5f. J. A. Lewis^[^ ^94^ ^]^ successfully realized high‐quality conformal printing of a rotating curved surface circuit by optimizing the structure of the printing nozzle, as depicted in Figure 5g. In addition, Park^[^ ^95^ ^]^ studied the extrusion printing technology of Ga–In–Sn alloy by changing the printing material type and improved the printing quality while maintaining high resolution, as shown in Figure 5h. J. A. Cardenas^[^ ^96^ ^]^ improved the electrical conductivity of the circuit surface through pulsed light ablation, as displayed in Figure 5i. R. Matsuzaki^[^ ^97^ ^]^ combined FFF with electroforming techniques to reduce the surface roughness of a printed circuit, as shown in Figure 5j. N. Lazarus^[^ ^98^ ^]^ reduced the resistance of the circuit connection through electroplating processing, which significantly improved the conductivity, as displayed in Figure 5k.

**Figure 6:**
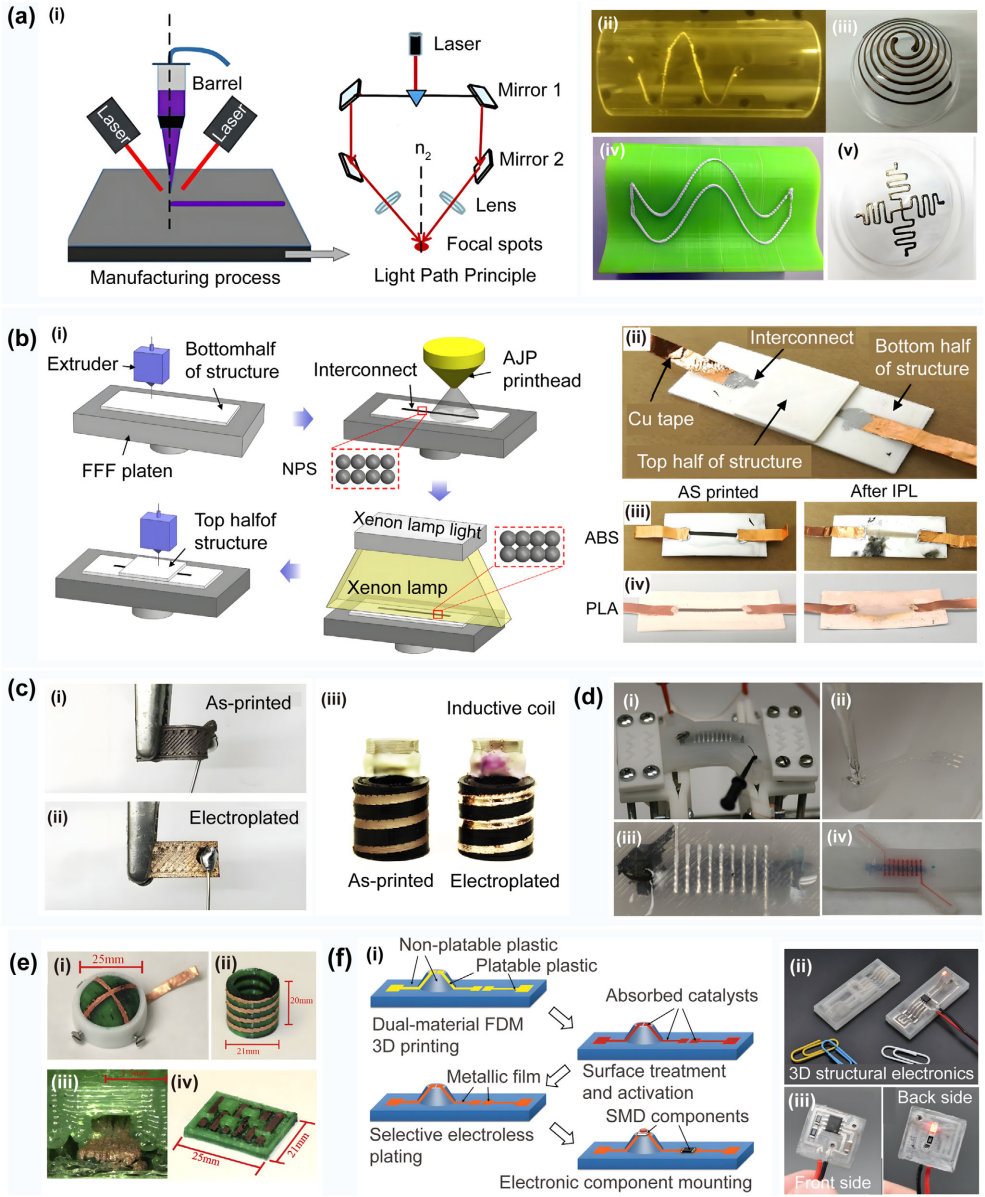
a) The design and implementation of a surface conformal 3D printing system: i) The direct‐write conformal printing system; ii) The conformal structure printed on the inner wall of the tube; iii) The printing result for the hemispherical conformal antenna before optimization; iv) The printing result for the free curve interpolation control process; v) The printing result for the optimized hemispherical antenna after optimization, Reproduced with permission from ref. [99] b) Flash light‐assisted additive manufacturing of 3D structural electronics. Reproduced with permission.^[^ ^100^ ^]^ Copyright 2022, ELSEVIER LTD: i) The structural print with embedded interconnections; 1) The FFF of the lower half of the structure; 2) The AJP of the interconnections; 3) The IPL; 4) The overriding of the upper half of the structure with the FFF; ii) Example of the fabricated structure and interconnection; iii) magazine of the ABS and PLA aggregates with the NW, with the NS ratio of 0:100. c) One‐step electroposition of the coupler on conductive 3D printed objects. Reproduced with permission.^[^ ^107^ ^]^ Copyright 2019, Elsevier: i) Picture of as‐printed structure; ii) Picture of electrified trains after confirmation; iii) Picture of as‐printed and electrified induced coils. d) The streamable inductor with a liquid magnetic core. Reproduced with permission.^[^ ^108^ ^]^ Copyright 2016, IOP Publishing: i) Inductor printing; ii) A liquid metal and a ferro fluid inductor filling; iii) After final fill with liquid metal and ferrofluid; iv) Filled with colored water to verify fluidic isolation. e) Lazarus, the selected electricity of 3D‐printed parts. Reproduced with permission.^[^ ^109^ ^]^ Copyright 2018, Elsevier: i) The region of casting for electricity; ii) Plated solvates, where the black sections denote the composition filament; iii) Coupler under tunnel; iv) Tunnel test piece. f) Hybrid additive manufacturing method for optional placing of free‐form circuit on the 3D printed plastic structure. Reproduced with permission.^[^ ^23^ ^]^ Copyright 2019, John Wiley & Sons: i) The process chain of the hybrid AM technology combining dual material FDM 3D printing and a selective electronics platform; ii) The 3D structural electronics; iii) The 2D double‐sided circuit board.

Once a circuit is printed directly onto an object's surface, high‐temperature sintering is required to achieve electrical conductivity. H. Liu^[^ ^99^ ^]^ studied the influence of the laser sintering process's parameters on the electrical conductivity of a circuit and successfully manufactured a hemispherical double‐helix conformal antenna with good electrical conductivity, which is presented in **Figure** **6**a. M. N. Jahangir^[^ ^100^ ^]^ significantly improved the electrical conductivity of a printed circuit by optimizing the intense pulsed light sintering (IPL) process's parameters, achieving a 300% increase in electrical conductivity, as depicted in Figure 6b.

However, although a sintered circuit has certain conductivity, its impedance is still high. Therefore, to improve the conductivity further, a common method is to replace the printing material or metalize the printed circuit surface.^[^ ^101^ ^]^ For instance, M.N. Jahangir^[^ ^102^ ^]^ used nanowires to replace traditional nanospheres, which significantly improved circuit performance.^[^ ^103^, ^104^ ^]^ The metallization treatment usually deposits a metal film on the printed conductive part by means of electrodeposition, which can both prevent the polymer material from melting during welding and provide a more stable electrical connection. Common metallization methods include electroless deposition and conductive coatings.^[^ ^105^, ^106^ ^]^ M. J. Kim^[^ ^107^ ^]^ demonstrated that using copper electrochemical deposition increased the conductivity of a circuit by 94 times and the ampacity by 17 times while reducing the surface roughness from 9.3 to 6.9 µm, as shown in Figure 6c. N. Lazarus^[^ ^108^ ^]^ concluded that copper‐electrodeposition technology could significantly improve the quality factor of a 3D printing solenoid coil, up to 1740 times, and increase the gain of a horn antenna by 1 dB, as presented in Figure 6d. Further, K. Angel^[^ ^109^ ^]^ reduced the inductor resistance by five orders of magnitude, from 3k Ω before plating, by selective plating, which is illustrated in Figure 6e. Li Ji^[^ ^23^ ^]^ significantly improved the reliability of circuit interconnection by selective metal plating on the surface of a 3D printing structure, making the resistivity as low as 6.7 × 10^−5^ Ω cm, as shown in Figure 6f. When the conductivity of a circuit is poor, the uniformity of electroplating can be affected by the voltage drop, resulting in uneven deposition of a metal film.^[^ ^110^, ^111^ ^]^ Accordingly, M. J. Kim^[^ ^107^ ^]^ explored the influence of a printing circuit's resistance on the uniformity of copper plating and found that the uniformity of deposit achieved by using electrified material was significantly better than that obtained with other low‐conductivity wires.

Although the direct printing process of a surface circuit enables fast manufacturing of surface circuits, both conductivity and mechanical reliability are still limited due to poor adhesion of the material between the conductive material and the molten substrate. The surface track filling printing technology can be used to optimize the material deposition and structural connection during the printing process, thus improving the conductivity and overall reliability of a circuit.

### 2.3 Surface track fill printing

**Figure 7:**
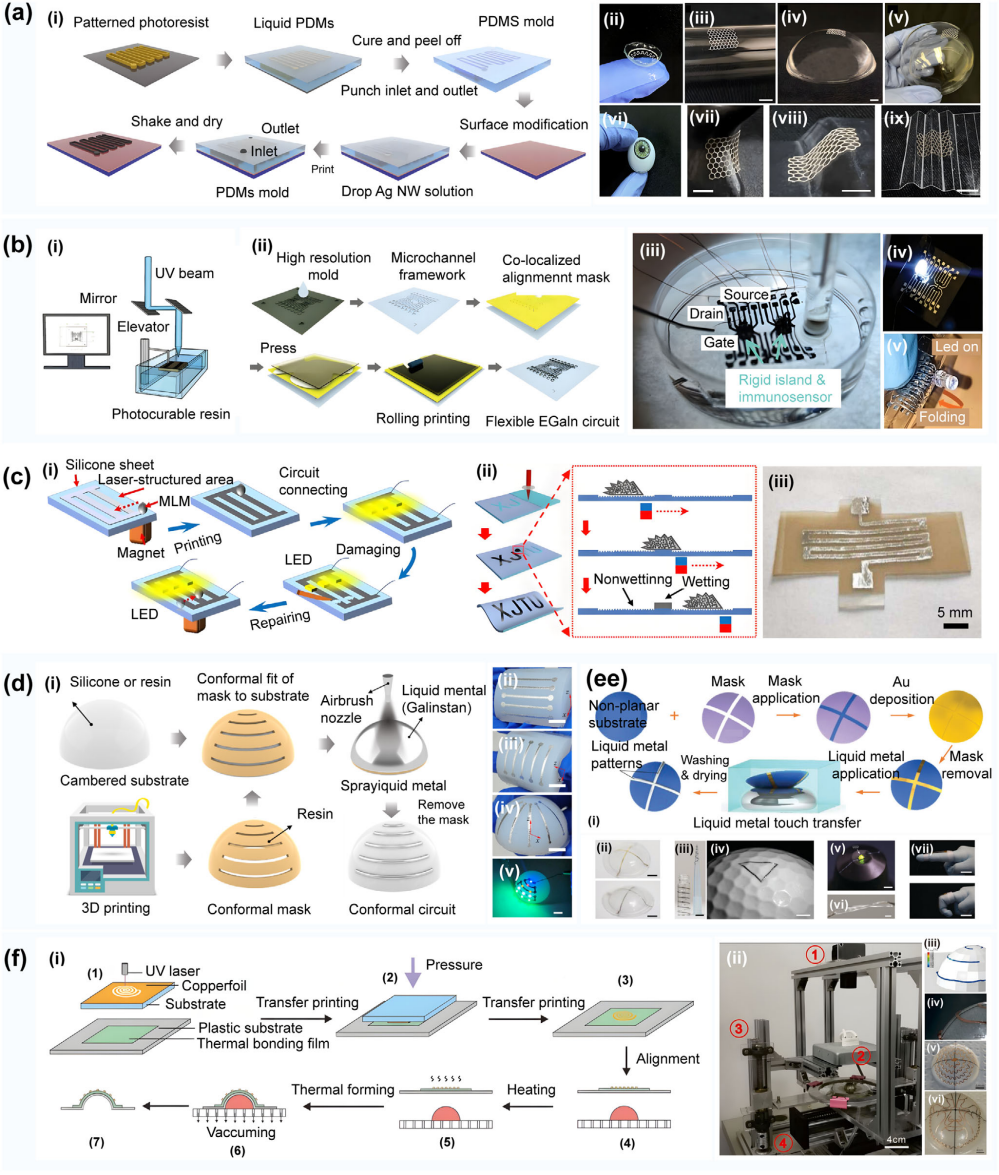
a) Curvilinear soft electronics obtained with the micromolding of metal nanowires in capillaries. Reproduced with permission.^[^ ^112^ ^]^ Copyright 2022, American Association for the Advancement of Science: i) A schematic diagram of the micromolding printing process; ii) Printed smart contact lens with a pressure sensor (serpentine pattern) and an antenna (circle); iii) The grid structure on a glass tube; iv) The hexagon grid structure on a PDMS hemisphere; v) The hexagon grid structure across the bottom and the side of a glass teacup; vi) Smart contact lens on an artificial eyeball; vii) Printed hexagon grid structures on a saddle‐shaped surface; viii) A pattern structure printed on a beveled surface; ix) Circuits printed on corrugated surfaces. b) Self‐healing electronics for the prognostic monitoring of methylated circulating tumor DNA Reproduced with permission.^[^ ^113^ ^]^ Copyright 2023, Wiley‐VCH: i) The projection micro‐stereolithography (PµSL)‐based high‐resolution 3D patterning process of an EGaIn circuit; ii) Aligned rolling printing of EGaIn circuits; iii) An overview of the iMethy device with an FET‐based biosensor; iv) Illustration of the functional self‐healing of the EGaIn‐based flexible circuit; v) The mechanical test of the EGaIn circuit. c) Guiding magnetic liquid metal for the flexible circuit. Reproduced with permission.^[^ ^117^ ^]^ Copyright 2021, IOP Publishing: i) Repair of a printed LM circuit by a magnetic field‐controlled MLM droplet; ii) Printing microstructures on the laser‐patterned surfaces; iii) Image of the printed LM circuit for the tensile sensor. d) Printing completely conformal liquid metal circuits on the arbitrary curved surfaces using a customized conformal mask. Reproduced with permission.^[^ ^114^ ^]^ Copyright 2024, John Wiley & Sons ‐ Journals: i) A schematic diagram of the process of liquid metal spraying on conformal circuits based on 3D‐printed CCMs; ii) Characterization of meridian circuits with different central angles on a half cylinder (line width: 1000 µm); iii) Characterization of radial circuits with different line widths on the half cylinder; iv) Characterization of radial circuits with different line widths on the half sphere. The scale bars indicate 10 mm; v) A multi‐colored LED array fabricated on a spherical surface using the fully conformal liquid metal process. e) Intermetallic wetting‐enabled high‐resolution liquid metal patterning for 3D and flexible electronics. Reproduced with permission.^[^ ^115^ ^]^ Copyright 2022, Royal Society of Chemistry: i) Non‐planar substrates using Au precursory patterns in an NaOH environment; ii) Au (top) and EGaIn (bottom) cross patterns obtained on a nonplanar plastic substrate; iii) Spiral liquid metal patterns on cylindrical (left, glass) and conical (right, plastic) objects; iv) Printed pattern structure on uneven plastic substrate; v) EGaIn transferred to a non‐planar surface of a 3D object, and the EGaIn track connected to an electronic component (i.e., LED); vi) Patterned long PDMS strips with Au orbitals; vii) Photographs of the relaxed state (top) and the bent/elongated state (bottom) of the EGaIn resistive motion sensor attached to a human finger in two different positions. f) High‐precision thermoforming 3D conformal circuit printing with a phase change adhesive layer. Reproduced with permission.^[^ ^118^ ^]^ Copyright 2019, MDPI. The manufacturing process of 3D conformable electronics: i) The major steps of thermoforming processing: 1) Patterning of the copper foil; 2,3) Transfer printing; 4) Visual alignment: The 3D model is put on the platform, and the sample is fixed in place with a special fixture; 5–7) Thermoforming to the target surface through vacuuming; ii) The overall structure drawing of the equipment; iii) The FEM results of circuit stress distributions; iv) Circuits at different heights; v) Printed physical image of the antenna circuit; vi) Direct manufacturing demonstration of the complex surface circuit pattern structure.

Surface mount circuits are constructed by reserving grooves on a surface as circuit tracks during the 3D model design phase and filling conductive materials after printing is completed, which finishes the construction of a curved surface conformal circuit. Y. Liu^[^ ^112^ ^]^ designed a specific miniature groove pattern template and successfully printed electronic circuits directly onto curved and corrugated surfaces, as shown in **Figure** **7**a.

As shown in Figure 7b,P. Fan^[^ ^113^ ^]^ constructed an EGaIn microfluidic surface‐mounted circuit with a self‐healing function by using projection microlithography technology, which can maintain conductivity under a 100% strain and realize self‐healing in case of severe damage. The high precision and high uniformity self‐healing surface circuit printing was achieved by filling in the reserved track with magnetic liquid metal, as depicted in Figure 7c. J. Chen^[^ ^114^ ^]^ combined 3D printing customized conformal mask and liquid metal inkjet technology, filled liquid metal into the mask groove of a curved surface substrate, and successfully constructed a liquid alloy curved surface circuit, as shown in Figure 7d. L. Johnston^[^ ^115^ ^]^ created a curved surface circuit with a resolution of up to 1.3 µm using the intermetallic wetting effect between an oxide‐free eutectic gallium indium (EGaIn) alloy and a high surface energy gold (Au) precursor layer, as presented in Figure 7e.

Similarly, Shi Peng^[^ ^116^ ^]^ proposed a circuit construction strategy based on the self‐healing characteristics of EGaIn; the constructed circuit resolution could reach 10 µm, and the oxide layer was manufactured in the PDMS microchannel by the roller printing method to seal the EGaIn effectively. Chen Feng^[^ ^117^ ^]^ introduced a technique for regulating the wettability of liquid metal using a femtosecond laser. K. Wu^[^ ^118^ ^]^ used a thermal phase change adhesive interlayer and an improved process, which significantly reduced the delamination and separation phenomena and improved adhesion, as shown in Figure 7f.

Recently, a variety of processes have been developed for the selective filling of conductive tracks on 3D‐printed surfaces to improve conductivity. Common methods include the direct writing method, the laser activation method, and a method for active material printing.^[^ ^119^ ^]^ A specific subcategory of these methods includes the silver spraying method,^[^ ^120^ ^]^ sputtering method,^[^ ^121^ ^]^ conductive spraying method,^[^ ^122^ ^]^ and electroless (chemical plating) method.^[^ ^123^, ^124^ ^]^ The direct write method is used to fill and form a complete circuit structure by depositing conductive ink in a reserved track for 3D printing.^[^ ^125^ ^]^ For instance, P.F. Flowers^[^ ^42^ ^]^ successfully created a 3D conductive line utilizing silver paste injection in 3D printed surface channels. However, although this method was effective in creating conductive tracks, the printed metal contact surface performance and electrical conductivity were still poor, and they were typically at least four orders of magnitude lower than those of block metal conductors.

**Figure 8:**
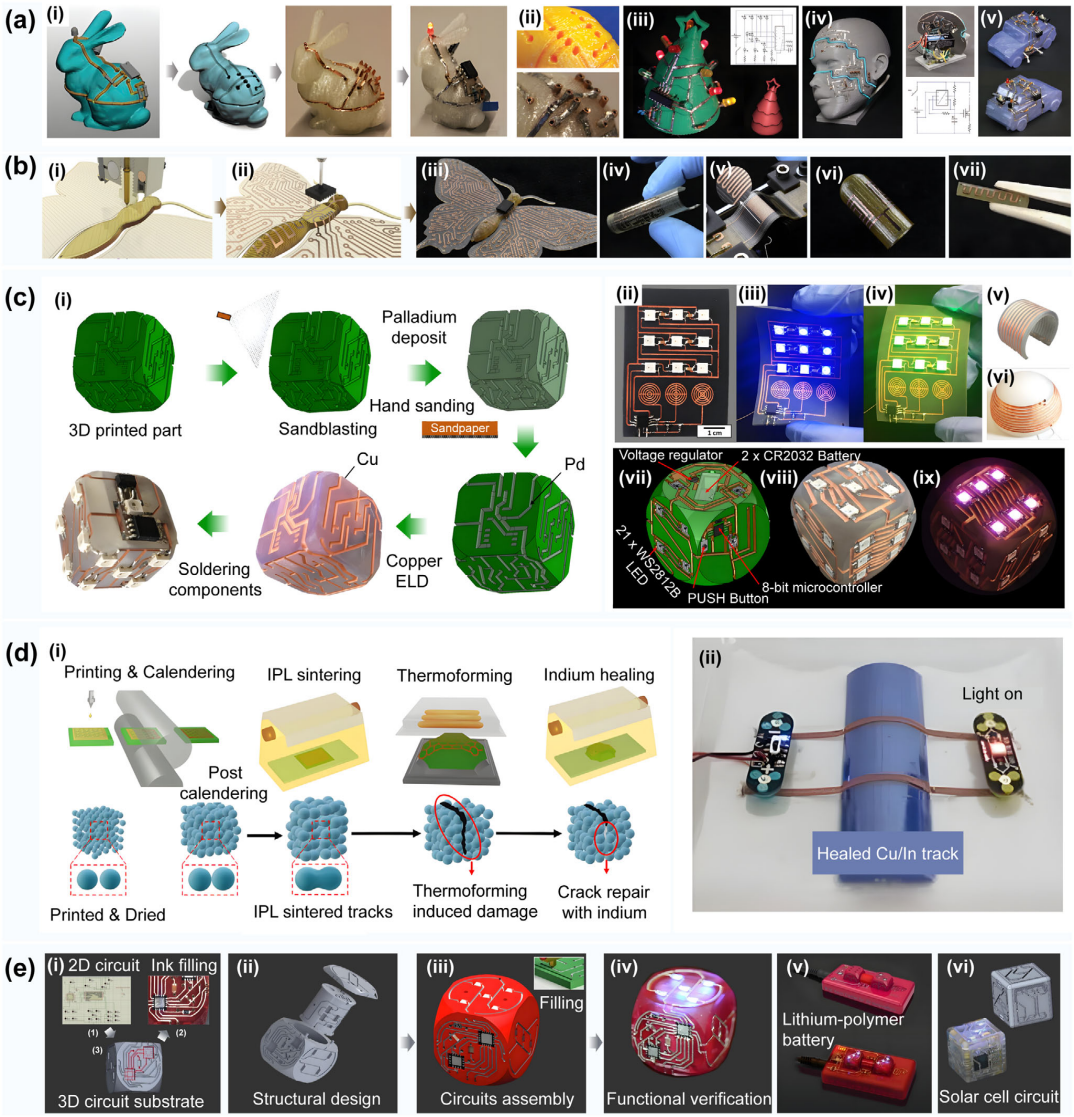
a) Surface mount circuits on 3D printed structures. Reproduced with permission.^[^ ^28^ ^]^ Copyright 2017, IEEE: i) Surface circuit workflow. The user first draws a 2D schematic diagram of a circuit and then 1) Positions the electrical components on a 3D shape and connects them with a curved trace; 2) The surface circuit automatically generates channels and holes on the surface; 3) To guide the user in the placement of copper tapes and tubes; 4) Finally, the user solders the copper pieces together. ii) An example of the channels and holes in (top) a 3D print and (bottom) the post‐soldering result; iii) The Christmas tree surface mount lighting circuit. The mounted circuits printed on the surface of a human face; iv) The surface mount circuits for automotive applications; b) The light‐based synthesis of metallic nanoparticles on the surface‐modified 3D printed substrates for high‐performance electronic systems, and a graphical schematic of the digitally driven fabrication process. Reproduced with permission.^[^ ^138^ ^]^ Copyright 2020, Elsevier: i) The fused filament fabrication of the PEI substrate; ii) Computer‐controlled solder paste being dispensed and surface mount assembly of electronic components; iii) A demonstrator manufactured using this photosynthetic technique, and the electronic‐packaging 555 timer with a powered flashing LED; iv) Highly ductile circuitry patterned on a 90 µm‐thick substrate; v) Combined rigid and flexible circuitry used to fabricate a positional sensor; vi) The fabricated pill capsule antenna sample; vii) Selective metallization of a 1.75‐mm diameter cylinder. c) Rapid 3D‐Plastronics prototyping with the selective metallization of 3D printed parts. Reproduced with permission.^[^ ^27^ ^]^ Copyright 2023, Elsevier: i) The processing steps: 1) 3D part with grooves printed using the SLA technique; 2) The surface physical modification achieved with sandblasting; 3) Colloidal palladium deposit on the entire surface; 4) Colloidal palladium localized only in the grooves after hand‐sanding; 5) Electroless Cu plating; 6) The final prototype, a flexible 3D plastronic device: ii) A photo of the device, iii, iv) The device operating in two bending positions; v) A picture of the manufactured 3D RTD sensor; vi) A spiral coil on a 3D‐printed half sphere using high‐temperature resin, and a gaming dice prototype: vii) The electronic circuit designed within the substrate; viii) The manufactured dice after 3D printing, metallization, and component soldering; ix) The functioning dice. d) Direct writing of self‐healing circuits on curvilinear surfaces. Reproduced with permission.^[^ ^139^ ^]^ Copyright 2022, EDITION COLIBRI AG: i) Self‐healable directly written circuits on curvilinear substrates; ii) LED demonstration with the Cu/In tracks. e) Embedded structure electronic manufacturing. Reproduced with permission.^[^ ^140^ ^]^ Copyright 2014, IEEE.

Aiming to improve the conductivity, the researchers have tried using copper with a lower resistivity as a conductive material instead of silver. For instance, G.T. Carranza^[^ ^126^ ^]^ effectively improved the conductivity of a circuit by attaching copper tape to the surface recess instead of using conductive silver paste. Since the connection between copper strips was realized by welding, and the melting point of solder was close to the melting point of the 3D printing plastic, the plastic would melt locally during welding, enhancing the mechanical bonding force of the circuit. As reported by N. Umetani,^[^ ^28^ ^]^ welding copper tape to the plastic could improve not only the electrical conductivity of a circuit but also its mechanical durability, as illustrated in **Figure** **8**a.

In addition to the method of replacing materials that fill conductive tracks, laser activation techniques have also been frequently used to improve the electrical conductivity of a circuit. The laser activation method is performed by depositing paint^[^ ^127^ ^]^ on a 3D‐printed fill track or by drip casting,^[^ ^128^ ^]^ spraying,^[^ ^129^ ^]^ and using a catalyst.^[^ ^130^, ^131^ ^]^ Recent studies have introduced several photopolymer materials suitable for the laser direct molding (LDS) process, such as Wenguang polymeric resins,^[^ ^132^ ^]^ photopolymeric resins containing lithium nepheline,^[^ ^133^ ^]^ and photocurable resins containing ZnO nanoparticles^[^ ^134^ ^]^ or tin oxide‐antimony.^[^ ^135^ ^]^ These photopolymer materials can effectively improve electrical conductivity through laser activation techniques. For instance, Semih Akin^[^ ^129^ ^]^ significantly improved the electrical conductivity of the circuit structure by atomizing a photopolymer coating in combination with laser activation techniques. S. Li^[^ ^136^ ^]^ successfully fabricated a conformal antenna using a laser activation process, which exhibited a quasi‐radiation pattern with a peak gain of −5 dBi. Although laser activation enables metallization of a circuit's surface, it is often associated with poor adhesion between the metallic coating and the plastic substrate. Therefore, to improve adhesion, pretreatment is often required for the surface to be plated to enhance the bonding strength between the conductor and the polymer interface.^[^ ^135^ ^]^ To this end, J. Yang^[^ ^137^ ^]^ selected synthetic copper aluminate as an activating filler on the resin surface, combined it with laser technology for surface activating treatment, and finally constructed a 3D circuit on the surface by chemical plating.

Similarly, selective deposition of high‐conductivity and high‐strength metallic conductors (e.g., copper,^[^ ^141^ ^]^ silver,^[^ ^142^ ^]^ gold,^[^ ^23^ ^]^ and nickel‐phosphorus alloy^[^ ^143^ ^]^) in the track by chemical plating (ELP) process can also enhance the electrical conductivity of a circuit.^[^ ^23^, ^144^ ^]^ For instance, P. Wang^[^ ^135^ ^]^ successfully constructed conformal curved surface 3D printing electronic products by combining photopolymerization (VPP) 3D printing and laser‐activated electroplated (ELP) technologies. In addition, J. Marque‐Hueso^[^ ^130^, ^145^ ^]^ manufactured copper conductors on FDM‐printed polyetherimide (PEI) surfaces by reducing the Ag + light layer into the polymer surface to the AgO layer as an electroless seed. J. Zhang^[^ ^146^ ^]^ successfully prepared a 3D line through the 3D printing of the acrylonitrile–butadiene–styrene copolymer (ABS) with or without a catalyst and combining it with chemical plating. N. K. Dixit^[^ ^147^ ^]^ successfully prepared a 3D circuit by processing the 3D‐printed ABS part surface with the epoxy resin paste and paint paste and conducting chemical copper plating. Although the method of chemical plating can effectively enhance the conductivity of a circuit, it introduces a risk of environmental contamination, which is toxic to humans and may involve carcinogens such as chromate.^[^ ^148^, ^149^ ^]^ Therefore, alternative safe treatment processes for chemical plating,^[^ ^150^ ^]^ such as plasma treatment,^[^ ^151^ ^]^ electroplating,^[^ ^152^ ^]^ and thermal spraying,^[^ ^129^ ^]^ have been proposed in recent years. Plating is an efficient method of metallization where metal ions are reduced by current and deposited onto a substrate's surface to provide uniform coverage of areas difficult to deeply metalize using conventional techniques.^[^ ^138^ ^]^ For instance, R.N. Esfahan^[^ ^138^ ^]^ successfully realized selective metallization of the PEI surface using the electroless process initiated by the local photo‐reduction of metal nanoparticles, which significantly improved the resistance of devices in a high‐temperature environment, as shown in Figure 8b.

Based on the 3D printing process of active material, the precision and effect of the metallization process can be significantly improved by directly applying a material loaded with an active chemical plating catalyst to the metallization track.^[^ ^146^, ^153^ ^]^ Research on light‐curing 3D printing has shown that this technology can effectively enhance the metallization accuracy of a circuit. R. Hensleigh^[^ ^154^ ^]^ proposed using an activated resin material with positive, negative, and neutral charges so that Pd catalysts with opposing charges can be accurately deposited locally at specific locations, thus improving the accuracy of circuit metallization. Related studies have also shown that the electrical properties of a packed track can be significantly improved by incorporating a catalyst into the resin material.^[^ ^139^, ^155^ ^]^ For instance, T. Gerges^[^ ^27^ ^]^ activated the photocurable resin with an adjustable ultraviolet light source and constructed a 3D circuit layer by layer through a reduction photopolymerization reaction, which significantly improved the circuit's electrical performance, as shown in Figure 8c. S.S. Park^[^ ^156^ ^]^ developed a self‐healing hybrid copper‐based ink that could be sintered and healed in milliseconds using an intense pulsed light printing circuit (Figure 8d). In terms of processing efficiency, E. Macdonald^[^ ^140^ ^]^ accelerated prototyping by applying 3D‐printed structural electronic components, shortening the development cycle time from weeks to hours (Figure 8e).

### 2.4 3D Printing without Welding

**Figure 9:**
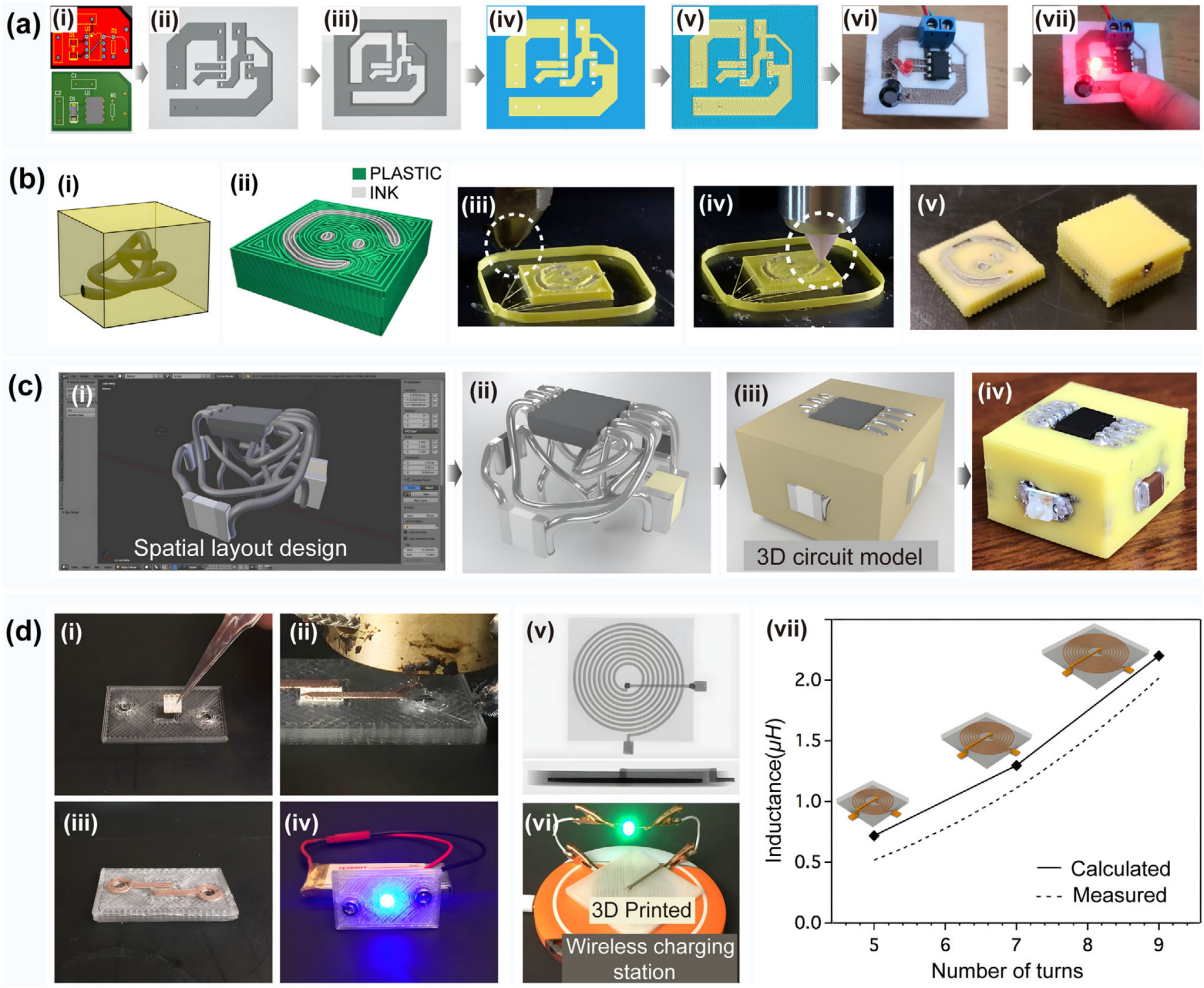
a) Direct printing strategy and application demonstration of a solderless stereo circuit. b) Automated hybrid 3D printing of 3D measuring interconnects. Reproduced with permission.^[^ ^157^ ^]^ Copyright 2019, IEEE; i) 3D design of a fastening electrical trace; ii) A cross‐sectional view of the dual‐filiation slicing of the preset line printing setup for multiple prematerials; iii) 3DP of the FFF; iv) 3DP of the microphones; v) Hybrid 3DP partial construction and full construction. c) Design and hybrid additional manufacturing of 3D/volume electrical circuits. Reproduced with permission.^[^ ^126^ ^]^ Copyright 2019, IEEE: i) A screenshot of the Brener 3D circuit tool environment; ii) The final 555 circuit model with components and interconnections; iii) Modeled 3D circuits; iv) 3D‐printed 3D circuits. d) 3D printing electronic components and circuits with conductive thermal insulation fittings. Reproduced with permission.^[^ ^42^ ^]^ Copyright 2017, Elsevier: i) Printing process is paused and LED is placed into a cavity before resuming printing. ii) Conductive traces are printed on top of the LED contacts, and iii) the LED component is fully embedded. iv) Screw terminals are used to attach a battery to the electrified traces and light the LED; v) A micro CT image of a 3D‐printed solar driver from the top‐view and side‐view perspectives; vi) Illustration of wireless power utilization of a nine‐turn solar inductor; vii) The construction process as a function of the number of turns.

The method of solderless stereo printing places a circuit structure inside an object and alternately deposits conductive and insulating materials to form a multi‐layer stereo circuit structure through 3D printing. Interface holes are reserved on an object's surface to insert components without welding.^[^ ^31^, ^32^ ^]^ This method includes the full 3D circuit modeling, circuit component layout, and wiring interconnection in a 3D environment. A typical manufacturing process is depicted in **Figure** **9**a. From design to manufacturing, the molding process of a solderless stereo circuit can be mainly divided into five steps: plane integrated circuit design, 3D circuit modeling, printing pretreatment, direct printing molding of a stereo circuit, and post‐printing circuit processing.

Generally, a solderless stereo circuit is printed using a dual sprinkler system, in which the left sprinkler is responsible for using the PLA‐shaped insulation circuit board and the right sprinkler is employed to print the conductive circuit structure using a conductive composite wire material.^[^ ^158^ ^]^ The formation of both the circuit board and the conductive circuit is completed in only one printing process with the cooperation of the dual sprinkler heads. When the main circuit body is formed through melt deposition, the electronic components are inserted and connected to the power supply for conduction, thus avoiding the welding step. U. Robles^[^ ^157^ ^]^ proposed a direct‐write 3D printing technique using a mixture of metal and dielectric materials. In this technique, the printed circuit was fully embedded in the dielectric and arbitrarily interconnected, as shown in Figure 9b. G.T. Carranza^[^ ^126^ ^]^ combined melt deposition molding and conductive silver paste micro‐spraying technology to achieve the solderless stereo printing of a 555 timer flashing LED circuit, as illustrated in Figure 9c,d. The free‐form manufacturing process of multi‐material stereo circuits proposed by Periard^[^ ^159^ ^]^ also demonstrated a certain potential for the construction of fully functional electronic devices. Patrick F. Flowers^[^ ^42^ ^]^ investigated the application potential of a dual‐material melt filament manufacturing process in a stereo circuit production and demonstrated the potential of manufacturing electronic components by using the FFF 3D printing techniques, as depicted in Figure 9d.

Although the solderless stereo circuit strategy achieves the fabrication of spatially curved circuits, the solderless stereo circuits comprise multilayer circuits stacked on top of each other, and the effectiveness of the interlayer bonding is dependent on the surface adhesion of the materials. Weak adhesion may lead to delamination and failure between the printed layers, especially when printing fine electrical traces.^[^ ^160^ ^]^ In addition, pulling and bleeding may occur during multi‐material 3D printing, leading to cross‐contamination between different materials, which may, in turn, trigger circuit shorts. Unlike solderless three‐dimensional circuits, embedded printing provides a more robust interlayer bond via internal soldering, which can reduce failures due to poor adhesion and materials interfering with each other.

### 2.5 Embedded Circuit Printing

Embedding electronic functionality in closed components offers many advantages, including a reduced number of parts and connections, a simplified assembly process, and a reduced weight. Embedded printing technology works by printing intra‐layer interconnecting conductive lines and inter‐layer interconnecting conductive lines in a predetermined path on the substrate while reserving a place for electronic components to be placed in the layer where the device is located. The thickness of the printed dielectric layer needs to be sufficient to ensure the complete encapsulation (isolation) of the intra‐layer interconnecting conductive lines in the component as well as its electronic components. Vertical interconnects that are electrically connected to the circuitry of the next layer must retain their tops to ensure an effective connection.

By embedding a 3D circuit into the model, the problem of poor adhesion between a circuit and a plastic substrate surface can be effectively avoided, and surface quality problems, such as the step effect, can be reduced while improving the overall printing effect.^[^ ^161^ ^]^ Currently, there are two embedding strategies: cavity mode and chamber mode. The cavity type of embedding strategy is also called the burying mode. In this strategy, the cavity is prefabricated on a 3D substrate, and then a conductive trace is made on the cavity's surface through a patterning process. Then, the SMD components are installed in the corresponding cavity positions, and the components and circuits are connected by a welding process. Next, the cavity is buried with a base photopolymer, and finally, a multi‐layer circuit is continuously printed with a functional photopolymer on a flat substrate. The chamber method omits the burying step by constructing the sidewall of a chamber directly on the circuit layer, then mounting the element on the bonding pad with silver paste, and finally, printing the dome or inclined roof directly and sealing the cavity with functional photopolymer. This mode requires careful design of the tilt angle of the cover to ensure the self‐supporting capacity.^[^ ^162^ ^]^

**Figure 10:**
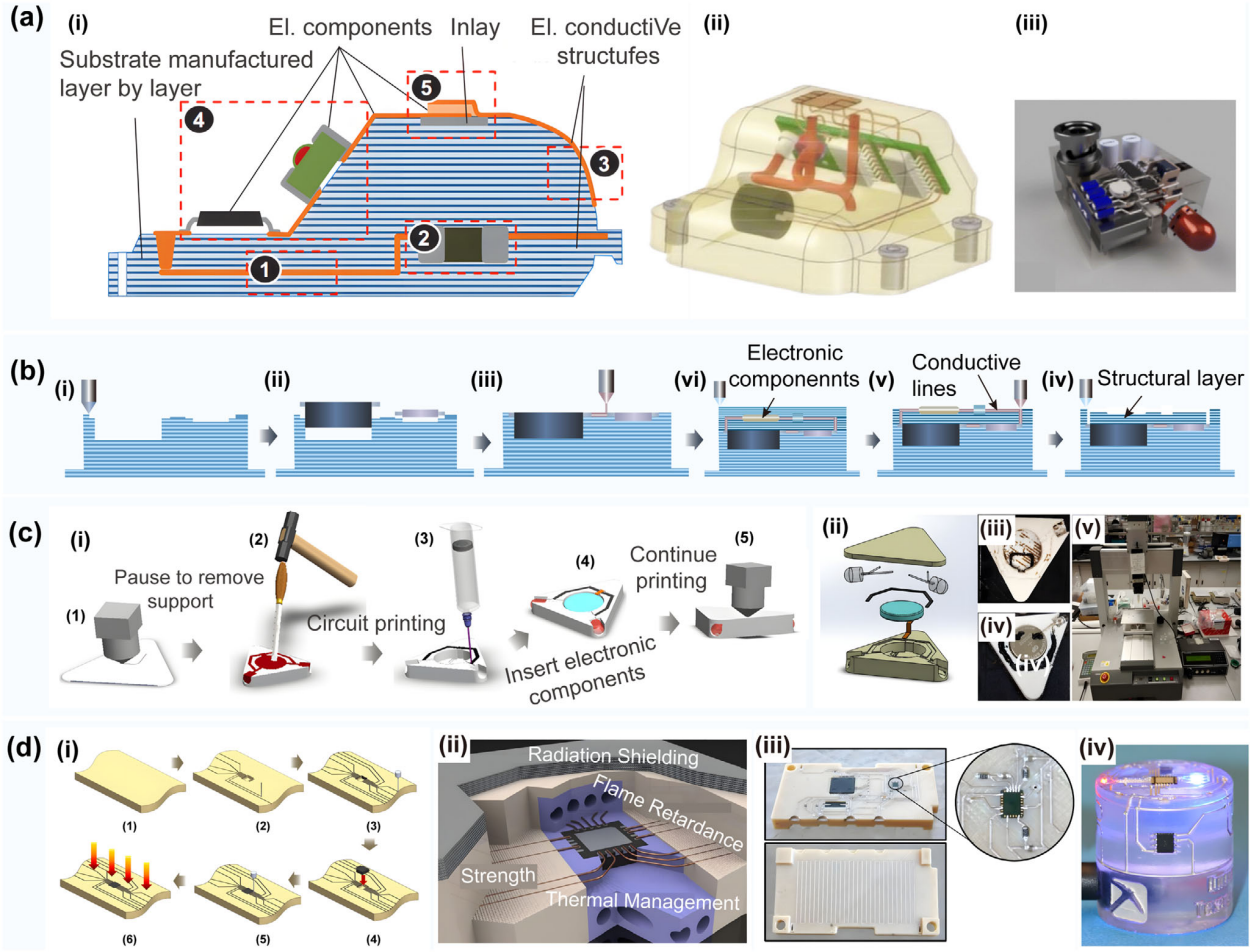
a) The manufacturing strategy for an embedded circuit: i) 1) Embedded conductive track; 2) Embedded electrical component and subsystem; 3) Printed 3D circuit track; 4) Mounted SMT components; 5) Electronic component printed on inlay; ii) The enhanced process of 3D printing, The embedding process of electrical components; 6) Connection of a component to the conductive path. Reproduced with permission.^[^ ^163^ ^]^ Copyright 2014, Elsevier; ii) Example of an eSLA module with integrated conductive circuits and electrical components. Reproduced with permission.^[^ ^164^ ^]^ Copyright 2014, Elsevier; iii) Examples of structural electronic equipment. Reproduced with permission.^[^ ^165^ ^]^ Copyright 2022, MDPI. b) Manufacturing process for embedded electronic devices. c) Additively manufactured multi‐material free‐form structure with printed electronics. Reproduced with permission.^[^ ^166^ ^]^ Copyright 2018, Springer‐Verlag London: i) The fabrication sequence of the free‐form structure; ii) A exploded diagram of the free‐form object showing various embedded electrical components, and images of the additively manufactured object showing iii) a semi‐fabricated part with removed support and iv) a structure with embedded components and printed circuit; v) The bioplotter system that is used to print electronic tracks in this study. d) The manufacturing strategy of the embedded stereo circuit. Reproduced with permission.^[^ ^167^ ^]^ Copyright 2014, Springer Nature BV: i) The processing steps of the 3D‐printed CubeSat module production using the FDM and conductive inks; ii) A schematic diagram of embedded stereo circuit fabrication; iii) The 3D‐printed CubeSat module produced using fused deposition modeling (the substrate material was ULTEM 9085), CNC routing, and direct print technologies; iv) Magnetic flux sensor system fabricated using a combination of surface mount circuitry and microelectronic components.

When selecting the embedding mode, the embedded type can improve the integration degree in the vertical direction for the components with a large size, and for small components, such as capacitance, resistance, and LED, the cavity type can save horizontal space.^[^ ^126^ ^]^ J. Hoerber^[^ ^163^ ^]^ successfully embedded electronic components into a 3D structure with multiple layers of circuitry by combining powder bed‐based printing techniques with the AJP techniques, as shown in **Figure** **10**a.

The embedded free‐form manufacturing process flow is shown in Figure 10b. G. L. Goh^[^ ^166^ ^]^ employed FDM and PolyJet printing techniques and printed electronic circuits within a structure to connect components. This procedure enabled the manufacturing of free‐form structures for embedded electrical components, as displayed in Figure 10c. Additionally, D. Espalin^[^ ^167^ ^]^ proposed a thermal embedding technique to immerse copper wires in a thermoplastic dielectric structure during an FDM process interruption. This technique allowed for the insertion of the conductors required for the electrical interconnection of electronic devices and components. Conductive inks were distributed in channels to provide electrical interconnection between the components, as shown in Figure 10d.

**Figure 11:**
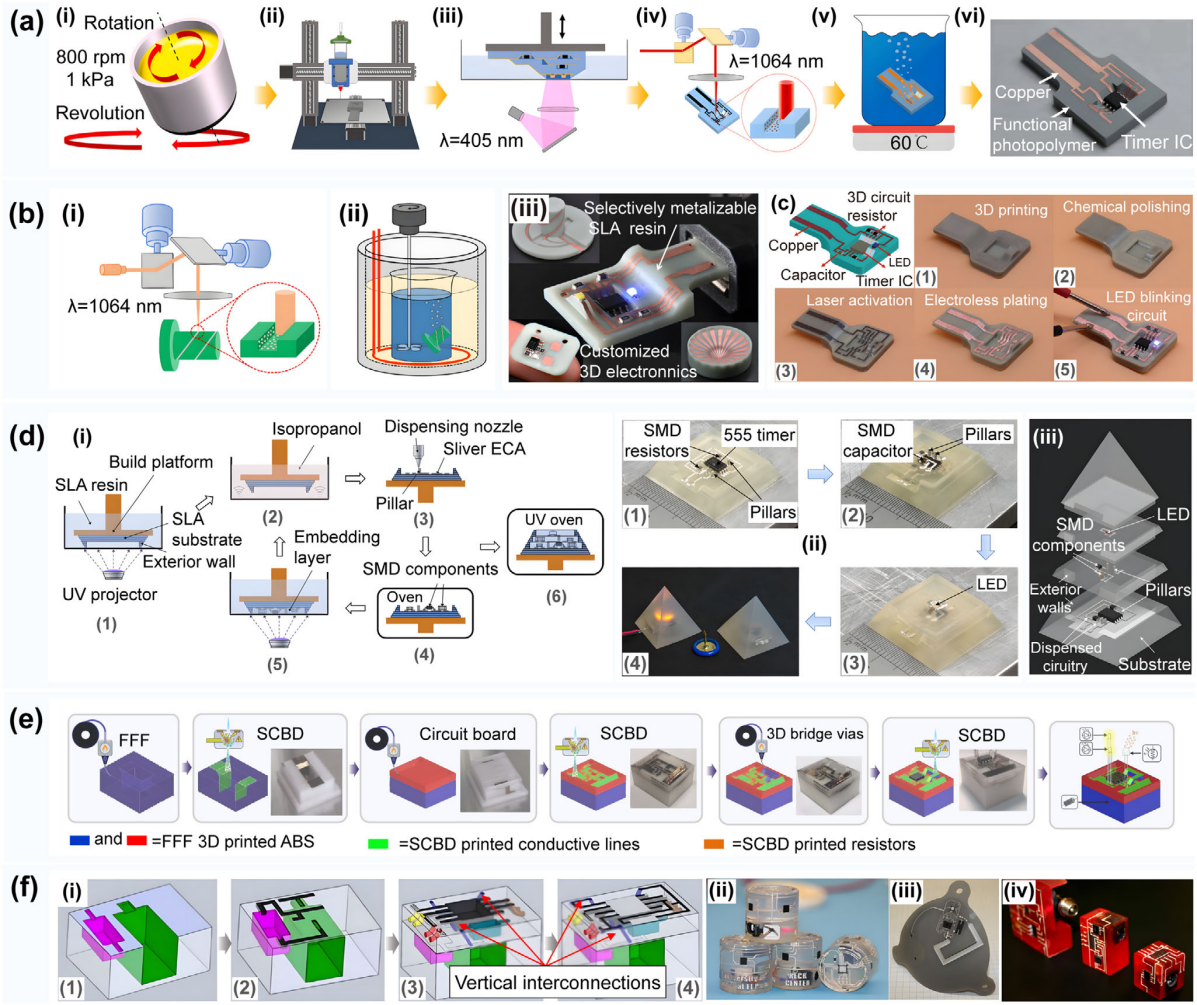
The embedded stereo electronic circuit manufacturing method: a) The process of enabling 3D multi‐layer electronics using a hybrid process consisting of vat photopolymerization and laser‐activated selective metallization. Reproduced with permission.^[^ ^26^ ^]^ Copyright 2023, Elsevier: i) Functional DLP photopolymer preparation; ii) DLP 3D printing; iii) Laser activation process; iv) Electroless plating process; v) Installation of electronic components; vi) The development process of a pyramid‐shaped LED blinking system with three embedded circuitry layers. b) Selectively metalizable stereolithography resin for 3D DC and high‐frequency electronics produced with hybrid additive manufacturing. Reproduced with permission.^[^ ^133^ ^]^ Copyright 2021, American Chemical Society: i) Laser activation process; ii) Electroless plating process; iii) Application of surface circuit to hybrid rapid forming technology; c) Laser‐activated selective electroless plating on 3D structures utilizing additive manufacturing for customized electronics^[^ ^119^ ^]^ John Wiley & Sons: The fabrication process of the 3D conformal 555 timer unstable oscillator circuit board (1, 5); d) Hybrid additive manufacturing of 3D electronic systems. Reproduced with permission.^[^ ^168^ ^]^ Copyright 2016, IOP Publishing: i) The process chain of the hybrid AM technology for 3D electronic system fabrication: 1) Substrate fabrication; 2) Ultrasonic cleaning; 3) Circuitry dispensing; 4) Component installation and ECA curing; 5) SLA embedding; 6) UV treatment; ii) The fabrication steps of the pyramid demonstrator: 1) The first layer circuitry; 2) The second layer circuitry; 3) The third layer circuitry; 4) The finished samples; iii) The exploded schematic showing the pyramid demonstrator; e) The process of embedding electronics in 3D printed structures by combining fused filament fabrication and supersonic cluster beam deposition. Reproduced with permission.^[^ ^173^ ^]^ Copyright 2018, Elsevier. f) Integrated stereolithography and direct printing for manufacturing 3D structure electronics. Reproduced with permission.^[^ ^179^ ^]^ Copyright 2012, Emerald Group Publishing Limited: i) Technological manufacturing process of a circuit: 1) The first SL build stop, with receptacles cleaned, and the first set of electronic components inserted; 2) SL to is continued embed components and build channels. The second SL construction stops, the channels are cleaned, the first DP process is conducted, and laser ink curing is achieved; 3) The SL construction is continued, the third SL construction stops, the receptacles and channels are cleaned, and the second set of components are inserted; 4) The second DP process and laser ink curing are achieved; ii) and iii) Examples of 3D structural electronics; iv)A three‐axis magnetic flux sensor system.

Vertical interconnection between different circuit layers is crucial to realize the multi‐layer system function in embedded multi‐layer circuit printing. P. Wang^[^ ^26^ ^]^ has proposed two vertical interconnection strategies: an internal strategy, where the upper and lower layers are connected through a burying or covering mode, and an external strategy, where the connection point of the circuit layer is led to the outer surface and the interconnecting wire is manufactured by laser activating the ELP, as shown in **Figure** **11**a. J. Li^[^ ^133^ ^]^ successfully fabricated high‐resolution conformal circuits on the free‐form surfaces by embedding them, combining three‐dimensional lithography (SLA) printing and laser‐activated chemical plating techniques, as illustrated in Figure 11b. P. Wang^[^ ^119^ ^]^ introduced a chemical polishing process based on acetone vapor, which could significantly improve the adhesion and electrical properties of the embedded copper layer, as shown in Figure 11c.

As depicted in Figure 11d, J. Li^[^ ^168^ ^]^ proposed a hybrid process that combined digital light projection (DLP) stereo lithography, 3D dot gum, and conventional surface‐mount packaging technologies in the manufacture of multi‐layer, multi‐material embedded electronic systems. W. Zhou^[^ ^161^ ^]^ combined melt deposition technology with inkjet printing of silver nanoparticle inks to produce embedded electronics and intelligent structures. Currently, most 3D embedded electronics construct embedded circuits by injecting conductive paste directly into microchannels for 3D printing.^[^ ^169^, ^170^ ^]^ However, this method is applicable only to continuous conductor circuits, whereas it is challenging to handle multi‐layer circuits of discrete conductors and electronic components.^[^ ^171^, ^172^ ^]^ In addition, the conductivity and mechanical strength of the conductive adhesive are lower than those of a metal conductor in traditional PCB technology, which limits the performance of the produced 3D electronic devices.^[^ ^33^, ^34^, ^43^ ^]^ To solve these problems, A. Bell^[^ ^173^ ^]^ employed a supersonic beam deposition (SCBD) technology to fabricate conductive embedded conductors^[^ ^174^, ^175^, ^176^ ^]^ and resistors^[^ ^177^, ^178^ ^]^, avoiding the application of welding and conductive adhesives to the production process, as shown in Figure 11e. A. Joe Lopes^[^ ^179^ ^]^ introduced a process that combines stereolithography (SL) with direct printing (DP) techniques for manufacturing embedded electronic circuits, as shown in Figure 11f.

**Figure 12:**
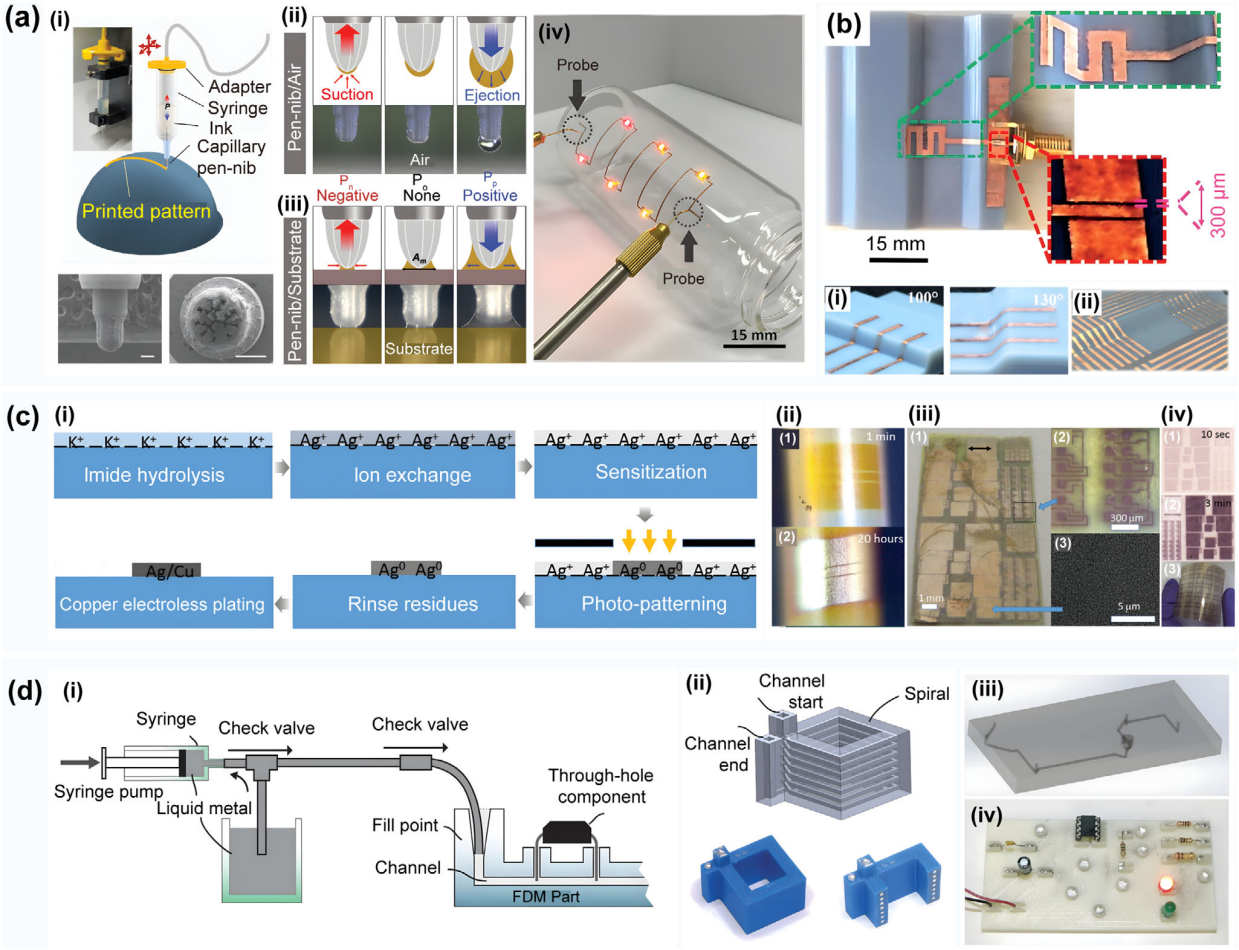
a) Air‐pressure‐assisted Pen‐Nib printing for 3D printed electronics. Reproduced with permission.^[^ ^182^ ^]^ Copyright 2022, John Wiley & Sons: i) A schematic illustration and photograph of the air‐pressure (AP)‐assisted pen‐nib printing head consisting of a capillary pen‐nib, syringe, and adaptor used to apply the air pressure, and the FE‐SEM images showing the side and front configuration of the nib (scale bar: 200 µm); ii, iii) The AP‐assisted control of the ink Qi formed on the front of the nib in ii) the air and iii) the substrate; iv) Illustration of conformal printing of a square‐wave‐shaped Cu interconnection; b) Binuclear copper complex ink as a seed for electroless copper plating yielding a bulk conductivity of more than 70% on the 3D‐printed polymers. Reproduced with permission.^[^ ^183^ ^]^ Copyright 2018, John Wiley & Sons: i) Copper lines on Veroblue with different angles; ii) A photo of a copper complex ink pattern inkjet printed onto a 3D‐printed object composed of a highly heat‐sensitive veroblue material; c) A rapid photopatterning method for the selective plating of 2D and 3D microcircuitries on polyetherimide. Reproduced with permission.^[^ ^130^ ^]^ Copyright 2018, John Wiley & Sons; i) The metallization process of polyimide and polyetherimide; ii) Polyetherimide ULTEM 9085 silver‐treated samples exposed at 460 nm; iii) The optical contrast of the photopatterned ULTEM 1000B silver and KCl treated samples; iv) 1000B ULTEM polyetherimide samples at different exposure times. d) Printing 3D electrical traces in additive manufactured parts for the injection of low melting temperature metals. Reproduced with permission.^[^ ^184^ ^]^ Copyright 2015, American Society Of Mechanical Engineers: i) Traces within the part having a sprue or injection point used to fill the trace and the outlet of the injection device seals into the injection port using standard slip tip syringe connectors; ii) A spiral‐shaped 465 mm‐long trace printed into a test part to illustrate the lengths at which the traces can be cast, and the ability to make 3D circuits; iii) Conversion of the circuit trace tree representation to the STL format; iv) The complete printed circuit system.

The continuity and stability of the interconnections are key prerequisites for establishing internal circuit connectivity in embedded electronic products. To construct 3D‐printed electronic devices with performance equivalent to that of PCB products, chemical plating (ELP) technology has been applied to the production of 3D embedded circuits.^[^ ^105^, ^180^ ^]^ There are two main processes in chemical plating: printing the dissimilar materials of the plating and non‐plating materials and depositing the metal layer on the plating part's surface.^[^ ^23^, ^144^ ^]^ For instance, J. Zhang and N Lazarus metalized only the plated portions through chemical pretreatment by doping ELP catalysts into the FDM material.^[^ ^146^, ^153^ ^]^ Another approach is to use selective surface treatment techniques, such as catalyst precursor printing, photolithography, or laser activation, to produce circuit patterns selectively and catalyze 3D printed surfaces, thus improving the circuit resolution.^[^ ^119^, ^133^, ^180^ ^]^ J. Ahn^[^ ^181^, ^182^ ^]^ metalized a circuit by printing an Ag catalyst ink on a 3D‐printed conformal structure, as shown in **Figure** **12**a. Y. Farraj^[^ ^183^ ^]^ created a near‐field communication antenna by spraying an ELP seed layer and selectively metalizing it on a 3D printing substrate, as presented in Figure 12b. J. Marque‐Hueso^[^ ^130^ ^]^ patterned the silver catalyst layer on the surface of polyetherimide (PEI) printed using the FDM using a photolithography technique, as displayed in Figure 12c. However, the ELP reaction occurred only on the catalytic surface, which limited the integration of the catalyst precursor printing.^[^ ^145^ ^]^

With the aim of improving the integration and enhancing the reliability of a circuit, P. Wang^[^ ^26^ ^]^ proposed a thermally active metal method, which combines the DLP 3D printing method with the laser‐activated ELP process. This combined method enables the manufacturing of a 3D electronic device with a multi‐layer circuit, where the conductivity of the ELP copper layer (5.2 × 10^7^ S m^−1^) is close to the conductivity of pure copper (5.8 × 10^7^ S m^−1^). J. P. Swensen^[^ ^184^ ^]^ designed a mechanical structure and a hollow channel part of an electronic circuit using the melt deposition molding technology and then filled the hollow channel with the liquid alloy to form a conductive circuit after solidification, as shown in Figure 12d.

By embedding a circuit into the internal structure of a product, the installation space can be saved, and the parts can be small‐sized, and also, the embedded structure can effectively prevent electronic components from being exposed to the external environment, thus improving their reliability. However, embedded circuits often require soldered connections during manufacturing, and high stresses at the soldered joints can affect, to a certain extent, their operation and integrity. In addition, the layer‐by‐layer stacking mode of an embedded circuit can also make heat dissipation and post‐maintenance more challenging. Circuit interconnection usually relies on a conductive via‐hole and bridge connection, which introduces great challenges to the multi‐layer circuit design.

### 2.6 In‐Mode Electronic Circuits

**Figure 13:**
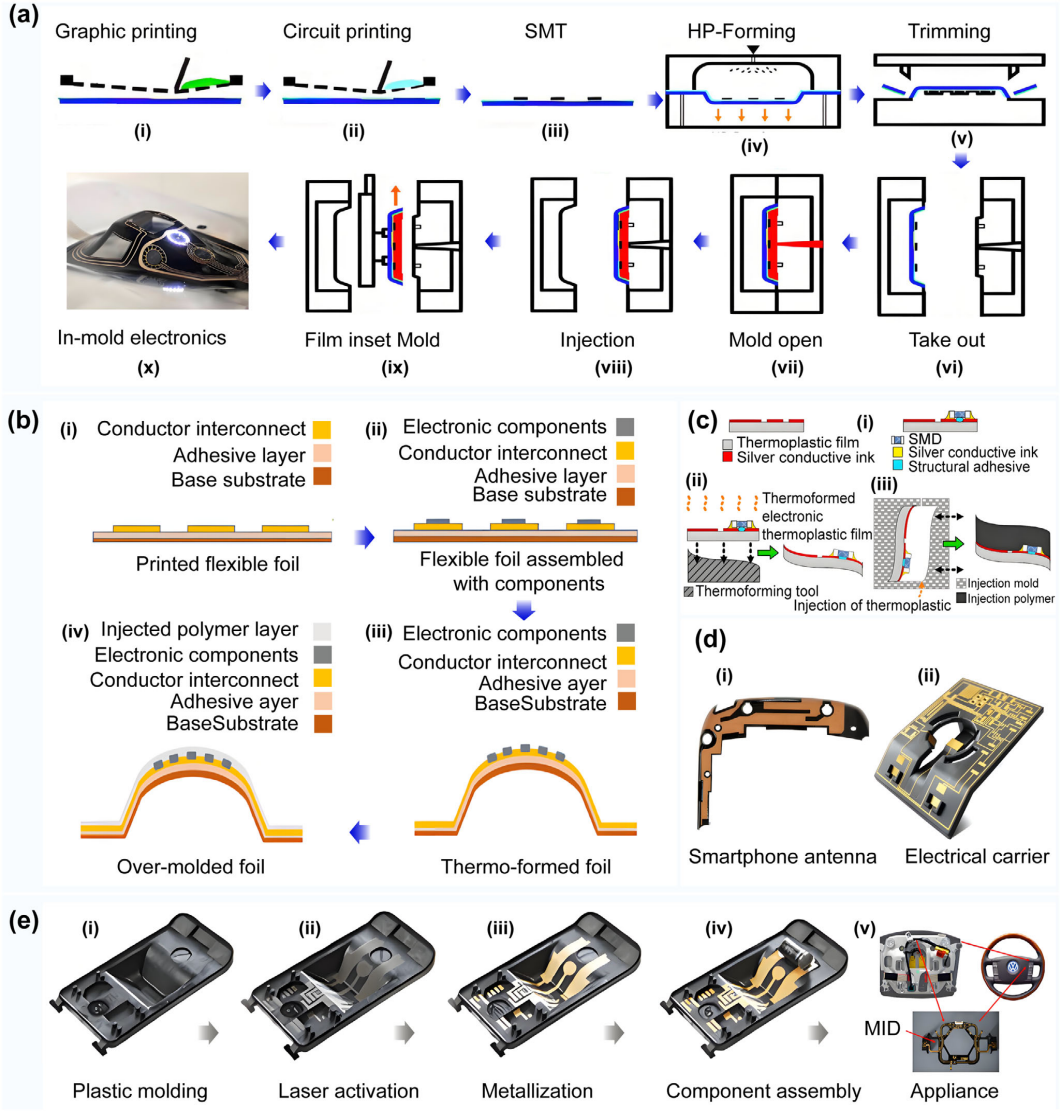
a) The complete process flow of the in‐mode electronics. b) Application demonstration of curved‐surface molding interconnection circuit. Reproduced with permission.^[^ ^29^ ^]^ Copyright 2022, IOP Publishing. c) IME manufacturing steps: i) screen printing of conductive ink and adhesion of SMDs, ii) thermoforming, and iii) overmolding by injection. Reproduced with permission.^[^ ^185^ ^]^ Copyright 2023, Springer Nature; d) The integration of electronic circuits in plastics using injection technologies. Reproduced with permission.^[^ ^29^ ^]^ Copyright 2022, IOP Publishing; e) The fabrication process of the laser direct construction (LDS) method: i) Plastic molding; ii) Laser activation; iii) Metallization process; iv) Component assembly; v) 3D MID used for reproducing the cable hardness of a sizing wheel. Reproduced with permission.^[^ ^30^ ^]^ Copyright 2018, Springer.

The IME technology first prints a conductive circuit structure onto a thermoformable substrate, then uses a conductive adhesive to secure the surface‐mounted components (e.g., surface‐mount devices and SMDs) to the circuit structure, and finally thermally forms the substrate to the desired curved surface shape by injection molding, as shown in **Figure** **13**a. In the process allowable range, the circuit structure can be molded into a complex curved surface shape, which can significantly improve the design freedom of the electronic product, as displayed in Figure 13b,c.

The functional materials used in the IME must have sufficient thermal forming resistance to withstand deformation during the forming process and maintain the stability of their electrical and mechanical properties. In the development process, the IME process usually encounters two major challenges: ink erosion and part warping and deformation. The main factors affecting these two types of defects include film thickness, injection temperature, injection pressure, and injection speed. Ink erosion is highly pronounced at small film thicknesses, especially near the gate, because the thin wall area near the gate generates large shear stress. Common solutions for erosion reduction include appropriately increasing the mold temperature, the injection melt temperature, and the melt injection pressure of the injection base polymer.^[^ ^186^ ^]^ Regarding part warping, a thicker film can exacerbate the hysteresis effect due to die temperature differences, causing non‐uniform temperature distribution in the die core and cavity, which results in residual stresses that can lead to warping. To solve this problem, recent studies have typically used measures of mold temperature reduction, injection speed increase, and injection pressure increase to improve the temperature distribution and reduce the warping effect.

However, the geometry of 3D‐MID products is often limited by the knockout process. The key development aspects of IME technology include material selection, circuit routing design, component layout, and optimization of molding process parameters. The production process requires that the material can withstand a 3D stretch, a high‐pressure stream impact, and thermal stresses generated during the hot forming and injection molding at high temperatures and pressures. Furthermore, as shown in Figure 13d, the integrated materials must be compatible to ensure adequate adhesion between the film and the injection matrix polymer.^[^ ^29^ ^]^ For instance, S. Balzerei^[^ ^187^ ^]^ studied using a preformed flame‐retardant glass‐fiber reinforced epoxy resin laminate (FR4) for back injection molding, which could significantly improve the effect of the injection molding process by optimizing the mold temperature, injection pressure, and pressure holding pressure. W. Kpobie^[^ ^188^ ^]^ studied the reliability and thermo‐mechanical behavior of an electronic board, analyzed the key parameters of the electronic forming process in the mold using the finite element simulation method, and found that the shrinkage rate, thermal expansion coefficient, and curing temperature had an important effect on the entire production process. Z. Wang^[^ ^189^ ^]^ examined the influence of different DCM contents on a printed circuit's conductivity and film‐forming capacity and conducted experiments with a conductive ink composed of nanosilver colloid, PVP/WPU, and thickener. X. Tang^[^ ^190^ ^]^ used grain‐free silver conductive inks as inks and PET films as substrates for in‐mold electronic manufacturing and found that silver film sintered bodies consisted of uniformly distributed nanosilver particles and that the film performance and resistivity varied with the number of printing layers. Although structural design reference data for IME products are limited, many researchers have been actively studying the standardization of this technology. I. Chtiooui^[^ ^30^ ^]^ described the key requirements for electronic packaging of IME, including the minimum contact distance, the minimum contact area, the overall height of components, and the humidity sensitivity level, as shown in Figure 13e. The IMSE design introduced by T. Simula^[^ ^191^ ^]^ can significantly reduce the weight (a 70% reduction) and thickness (a 90% reduction) compared to conventional multi‐component assemblies.

## 3 Curved Surface Circuit Applications

The advent of additive manufacturing technology has opened up innovative methods for the design and manufacture of electronic functionalized components. Through the embedded integration of conductive tracks and electronic components, the conformal printing of 3D curved circuits, the cross‐scale compatibility of surface mounting technology, and the collaborative manufacturing of multilayer heterogeneous structured circuits, this technology surmounts the spatial limitations of traditional planar electronic devices and achieves the conformalization of functions on complex curved surfaces. For example, in the field of health monitoring, curved surface circuits are able to closely fit with human tissues and collect ECG signals in real time.^[^ ^192^ ^]^ In the field of environmental sensing, 3D‐printed frequency‐selective surfaces can dynamically modulate the reflection phase of electromagnetic waves and reduce the radar scattering cross‐section.^[^ ^193^, ^194^ ^]^ This technology not only retains the high‐performance characteristics of planar integrated circuits but also significantly improves the mechanical stability and electromagnetic reliability of devices in extreme service environments.^[^ ^195^, ^196^, ^197^ ^]^ Currently, additive manufacturing techniques are widely used in smart wearable devices, curved conformal antennas, and smart skins for aircraft,^[^ ^181^, ^198^, ^199^, ^200^, ^201^, ^202^, ^203^, ^204^, ^205^, ^206^, ^207^ ^]^ and these techniques are expanding to more fields such as automotive electronics, biomedical, and energy systems.^[^ ^208^, ^209^, ^210^, ^211^ ^]^ **Table** **5** summarizes the applications and functions of curved conformal circuits.

**Table 5:** Comparison of applications and functions of curved conformal circuits.

Applications	Type of surface	Materials	Processes	Functions	Refs.
**3D antennas**	Hemispherical with convex and concave shapes	Silver ink	Microextrusion	Transfer signals	[94]
	Flexible paper with a coated layer	GaIn24.5‐based liquid alloy	Microextrusion	Transfer signals	[299]
	Gently curved	Aerosolized silver/Cu ink	Aerosol jet	Transfer signals	[300]
	Planar rolling material	Metal	Self‐assembly	Transfer signals	[301]
	Origami structure	Memory polymers and conductive foils	Light‐driven assembly	Transfer signals	[302]
	Adjustable spherical cap	Polydimethylsiloxane (PDMS) and liquid alloy	Elastic drive assembly and microfluidic	Transfer signals	[303]
	Hemispherical shape	PETG	Direct transfer	Transfer signals	[304]
	Spherical helix form	—	Pad‐printing	ESAs/display	[305]
**RF oscillator and transmitter devices**	Curved glass or flexible polyimide material	Viscoelastic silver‐nanoparticle inks	Microextrusion	Generate and process GHz signals	[25]
**Interconnection**	Glass material	Silver	Microextrusion	Connection	[306]
	Injection‐molded LCP	Aerosolized silver ink	Aerosol jet	Connection	[301]
	Cube	Liquid metal and polymers	3D printing	Transfer signals	[167]
**Stereo circuits**	Ceramic cube form	Silver ink in aerosol form	Aerosol jet	Enlighten	[9]
	Slightly curved Si	Silver	Omnidirectional printing	Provide light	[307]
	Stereoscopic substrate	Shape‐memory polymers	Thermally driven assembly	Enlighten	[22]
**LED arrays**	Curved ultrathin inorganic material	Semiconductor materials	Dual‐transfer method	Intravenous delivery systems	[211]
	Bendable material	Quantum dots	Intaglio transfer printing	Display	[308]
	Flexible/PI substrate	Au/In2O3 composite	EHD printing	Signal modulation	[309, 310]
**SWNT TFTs array**	Curved material	Carbon nanotube	Direct printing	Flexible and stretchable electronics	[311]
**Embedded sensors**	Curved or flexible glove‐like material	Liquid metal and ICs	3D printing	Sensing heat	[312]
**Fluid level sensors**	Polyhedron chamber tanks	Silver ink in aerosol form	Aerosol jet printing	Control and display circuits	[225]
**Gas sensors**	Curved design	Silver	Transfer printing	Test whether food has deteriorated	[313]
	Curved	PZT arrays	Transfer printing	Cutaneous pressure monitoring	[246]
**Wearable sensor**	Flexible PCB	Integrated array of chemical sensors	Photolithography	Multiplexed in situ perspiration analysis	[314]
	Tissue‐like polymeric substrates	TiO2 NM‐Au NPs‐TiO2 NM	Transfer printing	Diagnosis and therapy of movement disorders	[315]
	Flexible material	Lightweight sensor devices	Transfer printing	Skin display	[316]
**Sensor web**	Epicardium structure	Silk‐based substrate	Transfer printing	Electrophysiologic	[317]
	Rabbit epicardium	Functional metallic materials	Direct transfer	Spatiotemporal measurement	[318]
**Solar‐cell**	Slightly curved silicon material	Silver	Omnidirectional printing	Supply electricity	[307]
	Convex lens shape	Silver mesh	EHD printing	Heat supplement	[319]
**Metal‐network electrodes**	Versatile heat‐resistant glass shape	Silver mesh	EHD printing	Adjust the transmittance	[320]
	Curved polymer surface	Silver	EHD printing	Fabricating touch screen	[272]
**Epidermal electronics**	Curved material	Silver/sensors	Transfer printing	Monitor health	[321]
**Electronic membrane**	Curved material	Semiconductor wafer	Transfer printing	Flexible oxide TFTs	[322]
**Photodetector**	Dynamically adjustable hemispherical shape	PDMS	Elastic drive assembly	Electronic eye camera system	[322]
**Soft robot**	Arbitrary structure shapes	Ferromagnetic domains in soft materials	Magnetic‐driven assembly and 3D printing	Biomedical applications	[323]

### 3.1 Sensors

The applications of printed electronics have evolved from simple conductive layer paths, electrodes, antennas,^[^ ^212^, ^213^ ^]^ and RFID tags^[^ ^214^ ^]^ to more complex transistors and integrated circuits,^[^ ^215^, ^216^ ^]^ energy devices,^[^ ^217^, ^218^, ^219^, ^220^ ^]^ and sensors.^[^ ^265^, ^266^, ^267^ ^]^ The real‐time monitoring of surface‐related or environment‐related metrics can be achieved by printing sensors directly on non‐spreadable surfaces.^[^ ^221^, ^222^, ^223^ ^]^ These sensors are used in industrial applications (e.g., temperature and capacitive sensors for sensing touch^[^ ^224^ ^]^ or water level,^[^ ^225^ ^]^ as well as resistive sensors for pressure^[^ ^205^, ^226^ ^]^ strain monitoring,^[^ ^224^, ^227^, ^228^ ^]^ and structural health monitoring^[^ ^224^, ^229^ ^]^). Additionally, biochemistry plays an important role in being able to detect biosignatures,^[^ ^227^, ^230^ ^]^ humidity levels,^[^ ^231^, ^232^ ^]^ chemicals,^[^ ^233^ ^]^ and microbial concentrations,^[^ ^227^, ^234^ ^]^ which are important indicators for health diagnosis or food spoilage monitoring. For example, F. Wang^[^ ^1^ ^]^ fabricated hydrogel bioelectrode arrays for in vivo epicardial electrophysiological signal monitoring and electrical modulation using multi‐material DIW printing with conductive polymer hydrogel ink. G. Chen^[^ ^2^ ^]^ combined embedded 3D printing with biomineralization to successfully fabricate complex and mineralized free‐form structures and achieved this process without sacrificing ink properties. Y. Sun^[^ ^3^ ^]^ reported a ferromagnetic liquid crystal elastomer ink with 3D printed products capable of designable multimodal shape deformations with different stimuli. This ink has promising applications in wireless soft robots and smart devices. P. Zhang^[^ ^235^ ^]^ fabricated flexible electroluminescent devices and soft robots through a 3D printing technique based on direct ink writing. This technique could be used for artificial camouflage by displaying matching colors to instantly adapt to the environment and achieve artificial camouflage. M. Al‐Rubaiai^[^ ^236^ ^]^ proposed a compact and efficient stiffness modulation mechanism based on 3D‐printed conductive polylactic acid materials and demonstrated its potential in soft robots through stiffness and shape modulation.

### 3.2 Passive Components

The direct printing of passive electronic components on existing circuits can eliminate the interconnection and soldering steps common in the manufacture of traditional circuit boards.^[^ ^237^ ^]^ There have been several successful attempts to fabricate electronic components and devices with different multilayer multi‐material architectures via 3D printing, demonstrating the potential of this technology in electronics manufacturing. These devices include passive components (e.g., cross‐electrodes,^[^ ^238^ ^]^ antennas,^[^ ^239^ ^]^ resistors,^[^ ^240^ ^]^ and parallel‐plate capacitors^[^ ^42^, ^241^ ^]^) and multilayer circuits,^[^ ^242^, ^243^ ^]^ as well as active components (e.g., transistors,^[^ ^244^ ^]^ diodes^[^ ^214^ ^]^, and microelectromechanical (MEM) relays^[^ ^245^ ^]^), light‐emitting devices,^[^ ^246^, ^247^ ^]^ and energy devices.^[^ ^248^ ^]^ With 3D printing technology, components can be fabricated in a stacked fashion, thereby improving overall performance. For example, capacitors in stacked configurations exhibit capacitance values of 3.1 nF cm^−2^, which is higher than the 2 nF cm^−2^ value for a single capacitor.^[^ ^240^ ^]^ Flowers^[^ ^42^ ^]^ fabricated structured capacitors with capacitances as high as 150 pF using FDM printing technology. Hardin^[^ ^241^ ^]^ utilized a closed‐loop control scheme to fabricate high‐performance, fully printed high‐performance capacitors with capacitances up to 314 pF and breakdown voltages in excess of 1000 V, which is significantly higher than the standard for conventional printed capacitors. Correia^[^ ^240^ ^]^ fabricated printed inductors with an inductance of approximately 5.5 µH. These advances show the promise of 3D printing technology for passive electronic component fabrication.

### 3.3 Active components

Additive manufacturing technologies are gradually changing the way high‐performance active components are manufactured. Traditionally, these components have been produced using silicon wafer technology. However, using current additive manufacturing techniques, it has been demonstrated that various types of active components can be successfully and completely printed on a wide range of substrates. For example, Chen^[^ ^249^ ^]^ reported transistors that exhibited good mobility, excellent on/off ratios, and excellent current‐carrying capability through printing. Park^[^ ^245^ ^]^ fabricated fully printed relays using inkjet printing techniques. The device showed low on‐state resistance, very low off‐state leakage, and good switching speeds, demonstrating that additive manufacturing could produce high‐efficiency electronic switching devices. In terms of light‐emitting devices, Y.L. Kong ^[^ ^246^ ^]^ utilized extrusion‐based 3D printing to fabricate functioning LEDs for contact lenses that could emit light with a brightness of 100 cd m^−2^ at 4.5 V, and whose performance was comparable to that of LEDs fabricated on conventional planar substrates. Additionally, J. Zimmermann^[^ ^247^ ^]^ fabricated large‐area flexible light‐emitting devices on biocompatible substrates using hybrid printing techniques, further advancing the development of wearable electronics and flexible display technologies. Printed energy devices are still an emerging area of research compared to other electronic components and devices,^[^ ^250^, ^251^ ^]^ but progress has been made in the study of solar cells^[^ ^252^ ^]^ and piezoelectric energy harvesting devices.^[^ ^253^ ^]^ Currently, there are several approaches that attempt 3D printing with different material layers of solar cells.^[^ ^254^, ^255^ ^]^ For example, the aerosol jet printing technique has been used for the printing of the active layers of solar cells^[^ ^256^ ^]^ and photoelectrochemical cells^[^ ^257^, ^258^ ^]^ as well as the printing of current harvesting grids for solar cells.^[^ ^259^ ^]^ Recently, J. Luo^[^ ^260^ ^]^ reported a fully screen‐printed chalcogenide solar cell that could exhibit a short circuit current, open circuit voltage, fill factor, and power conversion efficiency of 22.8 mA cm^−2^, 0.911 V, 0.753, and 15.64%, respectively. This solar cell demonstrated that the fabrication of solar cells with 3D printing could achieve a considerably good performance. In the field of piezoelectric elements, M. Ali^[^ ^253^ ^]^ achieved voltage multiplication by printing interlayer connections between the electrodes of thin‐film piezoelectric elements, allowing all of the printed piezoelectric elements to be connected in series. Research in the field of energy storage devices is also advancing, with L. Liu^[^ ^261^ ^]^ reporting a fully 3D printed freestanding lithium‐ion battery and demonstrating the potential of 3D printing for battery applications. In addition, the research on micro‐supercapacitors has progressed.^[^ ^262^, ^263^ ^]^ L. Liu ^[^ ^264^ ^]^ fabricated a multilayer micro‐supercapacitor using screen‐printing technology that had a very high energy density (0.00433 mW h cm^−2^), remarkable flexibility (77.6% even after 1000 bending cycles), and cycling stability (10 000 cycles and still 82.6% after 10 000 cycles). This micro‐supercapacitor provided a new solution for energy storage in future flexible electronic and wearable devices.

### 3.4 Multi‐Material Printing

The use of multi‐material 3D printing technology in structural electronics is expanding. Three‐dimensional structures with multilayer circuits are produced by alternately printing conductive and insulating structural materials in each layer and connecting the different conductive layers through carefully designed interlayer conductive paths. A prominent example of coplanar multi‐material printing is the fabrication of three‐electrode electrochemical electrode systems.^[^ ^265^, ^266^ ^]^ Each electrode consists of a different material and is printed on the substrate separately. In addition, S. Li^[^ ^267^ ^]^ reported highly sensitive strain sensors prepared using aerosol jet‐printed electrodes and polyimide substrates for accurate strain monitoring. T. Hainsworth^[^ ^268^ ^]^ fabricated an actuator with embedded sensors using multi‐material printing. The actuator was designed to be safely integrated into human‐occupied environments to provide reliable support for robotic assistive systems, thereby demonstrating the potential of multi‐material 3D printing in the field of smart homes and robotics. In the field of architecture, J. Huang^[^ ^269^ ^]^ utilized freeform transformation techniques to enable the multi‐material additive manufacturing of conformal geometries, driving innovations in architectural structures and designs. E. Sowade^[^ ^270^ ^]^ combined conductive and non‐conductive materials into a multi‐material structure utilizing inkjet printing and screen printing techniques to fabricate components with 3D functional structures. J. Hoerber^[^ ^163^ ^]^ combined powder bed fusion and aerosol jet printing techniques to fabricate 3D circuit structures with multilayered multi‐materials. V. Correia^[^ ^240^ ^]^ presented a multilayered approach to the inkjet printing of resistors, inductors, and capacitors, providing fabrication materials and process steps for all passive devices.

### 3.5 Future Applications

In the future, additive manufacturing technology is expected to achieve higher integration, lightweight components, and intelligence in the fields of radar, antennas, detectors, and sensors, and to promote innovations in the military Internet of Things, big data analytics, and human augmentation technology. By printing circuits directly on equipment, this technology can significantly save space and improve the ability to adapt to complex environments. Additionally, in the future, customizable “smart” materials such as shape memory metamaterials, metal–organic frameworks, and supramolecular polymers will enable the precise programming of electrical conductivity and mechanical properties, leading to the development of multifunctional electronic systems. Moreover, curved circuit printing technology is particularly suitable for the fabrication of multilayered heterogeneous materials with high resolution and a wide range of electrical property modulation, which enables the printing of products with unsupported structures on complex curved surfaces.

In the biomedical field, customized bone repair scaffolds form distributed electrical stimulation circuits by embedding biocompatible electrodes into the pore structure of a scaffold with 3D printing technology. These circuits are capable of releasing electrical impulses to promote bone cell growth, avoiding the trauma associated with the secondary implantation of electrodes after surgery, and achieving the organic combination of therapy and structure. In the energy field, a curved photovoltaic‐energy storage integrated structure is used to embed flexible solar thin‐film circuits on the surface of wind turbine blades using 3D printing technology, and this structure is used to embed solid‐state lithium battery packs in the internal cavities of the blades to form a self‐sufficient energy system. This design makes full use of the curved surface of the blade's light energy collection area while avoiding the increase of additional wind resistance. The embedded energy storage unit and blade‐bearing structure conformal design effectively reduce the weight burden produced by the external battery compartment. In the field of robotics, the endogenous sensing network of a soft robot adopts multi‐material 3D printing technology to build spiral liquid metal circuits within a silicone substrate so that the circuits automatically stretch and maintain conductivity when the tentacles are bent. The circuit topology matches the robot's kinematic properties, enabling deformation‐adaptive force/tactile sensing, eliminating the risk of external cable entanglement, and enhancing operational reliability underwater or in a confined space.

The aerospace and automotive sectors have shown great interest in curved circuits, especially in miniature smart UAV applications. Traditional multi‐layer PCB circuit boards take up a large amount of space and weight, limiting the flight capability and payload of UAVs. Curved circuit technology, however, can embed circuits directly into the inner wall of a fuselage, significantly reducing weight and volume and thus increasing range and payload. This is pushing UAVs toward miniaturization, light weights, and longevity. At the same time, the integration of both antennas and circuits on curved structures can significantly improve communication and navigation capabilities and enhance a UAV's long‐range detection and stealth capabilities as well as maneuverability. In the field of military industry, the application of curved surface circuits can not only effectively reduce the weight of an aircraft but also improve the performance of the load and range and simplify the circuit system, thus reducing the probability of failure. Compared with the traditional riveting process, curved circuit technology can significantly reduce weight and fuel consumption. For example, a 15 percent weight reduction can increase a fighter jet's range by 20 percent and increase a payload by 30 percent. In addition, curved circuits can also be used to produce intelligent skins by using 3D printing technology to embed fiber‐optic sensor networks and strain‐monitoring circuits inside the aerodynamic surfaces of wings to achieve multi‐physical field in‐situ measurements and the real‐time monitoring of wing deformation and fatigue damage, thus improving flight safety. In the field of new energy vehicles, curved circuits can help reduce the weights of cables and circuits, increase specific power, and reduce fuel consumption, especially in the applications of battery status monitoring, sensors, and heat dissipation. At the same time, curved circuits can also provide vehicles with more accurate environmental awareness and intelligent features such as millimeter‐wave radar and intelligent in‐vehicle systems for enhanced safety and maneuverability.

## 4 Conclusions and Future Prospects

### 4.1 Challenges Faced by Curved‐Surface Circuits

Three‐dimensional‐printed circuit technology, as a cutting‐edge manufacturing method, still faces many issues regarding material properties,^[^ ^23^, ^35^, ^98^, ^107^, ^168^, ^184^, ^271^ ^]^ feature sizes,^[^ ^35^, ^184^ ^]^ fabrication efficiency,^[^ ^271^ ^]^ forming quality, and reliability,^[^ ^98^, ^107^ ^]^ despite its great potential for size optimization, performance enhancement, and system integration. Compared to printing on a flat surface, conformal printing on curved surfaces is more complex and involves more design and manufacturing considerations. For example, the printhead requires more degrees of freedom to access the surface of the 3D structure, more accurate tool path planning, and a larger amount of alignment of the 3D part with the printhead. To overcome these challenges, further technological innovations and engineering practices are required.

For materials, the types of conductive materials suitable for 3D printing are still limited. Although existing conductive materials, such as silver paste and carbon nanotubes, can meet the basic requirements for circuit manufacturing to a certain extent, the conductivity, stability, and long‐term reliability of these materials still need to be further improved. Currently, the conductivity of 3D printed circuits has not yet reached the level of traditional silicon‐based circuits, which limits their application in high‐precision and high‐performance electronic devices. Three‐dimensional structured electronic devices usually involve a combination of multiple materials such as conductive, insulating, and semiconductor materials. The compatibility of conductive materials with other materials during multi‐material 3D printing has still not been adequately addressed. In particular, in most cases, the conductive layer is composed of metals, while the dielectric layer is usually composed of polymeric materials. The mismatch between the expansion coefficients of these materials usually leads to high residual stresses in the film, which in turn triggers interfacial delamination that may lead to structural instability, failure, or even cracks. To solve this problem, the bonding strength at the interface needs to be enhanced with gradient transition layers or flexible cushioning structures to relieve the stresses generated by the difference in the coefficients of expansion for the different materials. Ensuring good bonding between different materials is the key to safeguarding circuit stability and performance. Hence, determining how to improve material compatibility and interface strength is still an important challenge that needs to be overcome by current 3D printing circuit technology.

For printing accuracy, the precision of 3D printed circuits is limited by the resolution of the printer. Despite the progress made by high‐resolution printers in certain applications, their resolution is still not comparable to that of traditional silicon‐based microelectronics when fabricating high‐density and micro‐sized circuits. In 3D stacked structures, the fabrication process requires a high degree of precision since components are embedded within the three‐dimensional structure body or arranged on the surface of the substrate with the shape. In particular, in the fabrication of multilayer circuits, fine wires, micro‐components, and complex curved circuits, there are still many challenges for high‐precision and high‐quality manufacturing. Specifically, the alignment between layers must be very precise, and any small error may affect the performance and reliability of a device. To ensure the precision and performance of circuits, further optimization of printing technology is needed to improve printer resolution and accuracy, and more advanced manufacturing processes are needed to address these precision and quality control issues. Specifically, in the production of curved circuits, determining how to solve these challenges and ensure the continuity and surface flatness of the circuits will be a key direction in the development of 3D printed circuit technology.

In application circuits, in addition to ensuring good electrical conductivity, the stability and long‐term reliability of a circuit are also key issues, especially in high‐power application scenarios. Determining how to effectively manage the circuit heat dissipation to prevent overheating and damage is still an urgent research topic. The non‐planar geometric features of 3D structures (such as curved surfaces, overhanging layers, and heterogeneous cavities) significantly enhance the multi‐physical coupling effects of electromagnetic field distribution, thermal conduction paths, and mechanical stress fields. In 3D structures, the density of the electronic components is high, and the stacked design will exacerbate the thermal gradient and lead to localized heat buildup, which may trigger problems such as electromigration and thermal runaway, or even cause chip failure. Therefore, the adoption of microfluidic cooling or high thermal conductivity interface materials (e.g., graphene/aluminum nitride composites) and thermal management design through topology optimization, thermal analysis, and simulation with comprehensive consideration of multi‐physical field effects (e.g., thermally induced warpage and mechanical stress) has become one of the solutions used to balance the electrical‐thermal‐force performance. In terms of mechanical performance, the strength of 3D printed circuits is usually lower than that of conventional circuits, especially in curved or complexly shaped circuit structures. Determining how to ensure the durability, bending, and compression resistance of circuits remains a challenge that must be addressed. For component assembly and circuit connections, interlayer connections, soldering, and assembly issues need to be considered. In 3D structures, different layers need to communicate with each other through vertical electrical connections, e.g., through miniature conductive vias or three‐dimensional interconnections, which require high‐precision fabrication techniques and reliable connection points. In 3D devices, the soldering and assembly process is even more complex, and it must be ensured that multiple layers of components can be accurately connected in a limited space while guaranteeing the quality and stability of the soldering points.

In addition, determining how to ensure the consistency and reliability of printed circuits through effective quality inspection means is also one of the current technical challenges. For the testing and verification of electrical functions, surface circuit structures are relatively easy to test, but for multilayer nested three‐dimensional circuit structures, testing the performance of each layer becomes more difficult. Since traditional test methods may fail due to the difficulty of direct access to certain parts, specialized test architectures and methods need to be developed, such as the embedding of test circuits in a design to ensure that the functionality and performance of each layer meet the requirements. Finally, the environmental adaptability of 3D printed circuits for long‐term use for environmental factors such as humidity, temperature variations, and chemical corrosion remains to be addressed. Changes in temperature and humidity may lead to a redistribution of metal particles in the thermoplastic material, thus affecting the conductivity and stability of the circuit. Therefore, future research needs to develop new testing standards and quality control methods to improve the reliability of circuits under different operating conditions, especially in extreme environments.

For the manufacturing process, although multi‐axis linkage technology enables the conformal printing of curved circuits and the embedded fabrication of 3D circuit structures, it still faces many challenges in practical applications. First, it is very difficult to print materials on concave surfaces with large curvature. It is difficult for the block nozzles used in 3D printing to work efficiently in narrow spaces, which in turn affects the feasibility of printing. Second, the multi‐dimensional layout of circuits needs to take into account the design complexity. Curved circuits require layout designs in three dimensions, as well as the rational placement of the circuits and components through synergistic interconnections between different layers, ensuring that the circuits and components do not interfere with each other. In addition, 3D circuit structures typically have higher voltage drops than planar devices, and vertical interconnects are more susceptible to signal attenuation and crosstalk than planar interconnects, which may lead to signal delays. Therefore, it is particularly important to optimize the interconnection topology (e.g., mesh or a tree structure) as well as the design of the power supply network or dynamic voltage regulation for multi‐layer structures. During high‐frequency signal transmission, vertical vias may have higher capacitance than planar wiring and thus may need to be optimized with shield design or impedance matching to ensure signal integrity and timing performance. Another challenge is that because 3D printing uses layer‐by‐layer stacking, there can be a significant “step effect” during the printing process, which can lead to uneven circuit surfaces, thereby affecting conductivity and overall performance. In addition, the conductive filaments used in 3D printing typically have high resistivity and poor conductivity, making it difficult to meet the demands of high‐performance circuits. The poor adhesion of conductive materials to the printed plastic surface is also a significant issue. Current technology relies on conductive adhesives or low‐temperature bonding materials, but these methods are prone to warping, peeling, or breaking due to vibration or external forces, significantly reducing the reliability of a circuit. Finally, the problem of connecting circuits to electronic components cannot be ignored. Since thermoplastic conductive filaments tend to melt at high temperatures, they cannot be connected using conventional soldering, which reduces the reliability of the connections between electronic components and further restricts the feasibility of 3D printed circuits in high‐reliability applications. Therefore, to address these challenges, future research should focus on increasing the electrical conductivity of printed materials, improving material adhesion, optimizing circuit layout design, and exploring new connection methods to promote the practical application of 3D printed circuit technology.

Although 3D structured electronics face a series of challenges in design and fabrication, such as geometric complexity, multi‐physical field coupling, and process compatibility, their unique advantages in high‐density integration (volume reduction), multi‐functional fusion (e.g., integration of sensing, energy storage, and communication), and spatial adaptability (e.g., wearable devices) make them irreplaceable in the fields of aerospace, biomedicine, and biomimetic robotics, among other application prospects. These challenges need to be addressed using advanced design tools, innovative design methods, and improvements in manufacturing processes. To address these issues, interdisciplinary collaboration (e.g., the convergence of materials as well as mechanical and electrical engineering) is particularly important. At the same time, technological innovations, such as the application of quantum dot 3D printing technology and self‐healing interfaces, offer new directions to surmount existing bottlenecks. Utilizing these innovations and collaborations, the full potential of 3D devices in terms of high integration and performance can be unleashed, driving their practical applications in various fields and enabling broader technological advancement and industrialization.

### 4.2 Summary and Future Prospects

The emergence of 3D‐printed curved circuit technology has broken through the limitations of traditional circuit manufacturing processes, making it possible to integrate circuit structure and function to a high degree. This technology combines the advantages of additive manufacturing to provide innovative solutions for electronic products in terms of miniaturization, light weight, intelligence, and personalization. By employing 3D printing strategies, the fabrication of non‐planar surfaces or embedded constructed components becomes possible, driving new design concepts while simplifying the production process and providing new paths for personalization. This paper summarizes the latest research progress in 3D printing curved circuit technology in terms of materials, processes, applications, development prospects, and challenges faced.

Currently, the main methods for manufacturing curved circuits based on 3D printing technology can be classified into two categories. The first category involves integrating conductive circuits into the interior of an object, and the other category involves printing conductive circuits directly onto the surface of an object. Although there are existing technologies that can achieve the printing of curved circuits, these technologies still face many challenges when dealing with complex shapes, especially large curvature concave surfaces and multi‐material substrates. Specifically, issues such as material selection, precision control, and structural reliability remain problematic when producing curved circuit structures with high resolution, high conductivity, and high mechanical strength. Ideal 3D‐printed electronic functional materials need to have excellent electrical properties and material characteristics as well as good printability. These two factors directly affect the final electrical performance and print resolution, so they are crucial in the design of 3D‐printed electronics. Although there is still a gap between current 3D printed circuit technology and traditional silicon‐based microelectronics in terms of performance, it has significant cost advantages, especially in the manufacture of large‐area, lightweight, and miniaturized electronic products. Additionally, 3D printing technology demonstrates a unique market advantage that cannot be replaced with silicon‐based microelectronics.

Artificial intelligence (AI) and machine learning (ML)‐assisted 3D topology generation algorithms and self‐healing interface materials can be explored in the future to break through the existing technical bottlenecks. Possible development directions include circuit layout design optimization, manufacturing process improvement, new material development, and print quality control and testing. In terms of design optimization, machine learning algorithms can predict the best structural parameters to generate lightweight and highly thermally efficient 3D structures, thus improving thermal management performance and signal integrity. At the same time, these algorithms can be used to simulate and predict the performance of 3D devices under different operating conditions, which helps designers identify potential problems in advance. For manufacturing process improvement, AI can be used to analyze manufacturing data to optimize 3D printing parameters such as print speed, temperature, and material ratios, to improve print accuracy and reliability. Machine learning can also be used to monitor and adjust the printing process in real time to reduce defects and improve yields. In terms of material development, AI can be used to accelerate the research and development of new 3D‐printed electronic materials, predict material properties, and guide experimental design through machine learning, which includes conductive ink, thermal interface materials, and structural materials. In terms of quality control and testing, AI is used to automatically detect defects in 3D printed electronics with the help of image recognition and data analysis and to make self‐adjustments based on real‐time feedback to optimize the efficiency of quality control. At the same time, machine learning is used to analyze test data to predict the long‐term reliability of a device.

The main purpose of using printed electronics is to provide an alternative to existing options, not a complete replacement for existing printed circuit technology or passive and active components such as silicon semiconductor structures. This technology is intended to be complementary to existing technologies and to address certain needs that cannot be met with traditional methods. Currently, printed circuits offer lower performance, especially in integrated circuits operating at high frequencies. Silicon semiconductor chips remain important in electronic solutions for large‐scale integration and high‐performance requirements. However, the innovative and forward‐looking nature of 3D printed curved circuit technology has made it the focus of global research and industrial applications, and this technology has been highly recognized by many related industries. A greater number of specialized fields have begun to draw on the principles of printing technology to solve technical challenges, and significant progress has been made.

Overall, 3D‐printed curved circuits have proven to be a viable and innovative solution for circuit manufacturing. The printing of circuits directly onto the interior or surface of a product not only achieves structural and functional integration but also significantly improves space utilization, reduces weight, enhances integration, and improves circuit layout flexibility. This technology provides greater design freedom for all types of applications while improving overall performance. As curved circuits with integrated functional systems continue to evolve and mature, the potential of 3D printed electronics in a wider range of application scenarios will further drive the innovative application of printing technology in a number of industries, demonstrating its powerful technical solution capabilities.

## Conflict of Interest

The authors declare no conflict of interest.
